# Determination and characterization of molecular heterogeneity and precision medicine strategies of patients with pancreatic cancer and pancreatic neuroendocrine tumor based on oxidative stress and mitochondrial dysfunction-related genes

**DOI:** 10.3389/fendo.2023.1127441

**Published:** 2023-05-08

**Authors:** Yougang Cui, Qihang Yuan, Junhong Chen, Jian Jiang, Hewen Guan, Ruiping Zhu, Ning Li, Wenzhi Liu, Changmiao Wang

**Affiliations:** ^1^ Department of General Surgery, The First Affiliated Hospital of Dalian Medical University, Dalian, Liaoning, China; ^2^ Department of Gastrointestinal Surgery, The Affiliated Zhongshan Hospital of Dalian University, Dalian, Liaoning, China; ^3^ Department of Hepatobiliary and Pancreatic Surgery II, General Surgery Center, The First Hospital of Jilin University, Changchun, China; ^4^ Department of Dermatology, The First Affiliated Hospital of Dalian Medical University, Dalian, Liaoning, China; ^5^ Department of Pathology, Affiliated Zhongshan Hospital of Dalian University, Dalian, Liaoning, China; ^6^ Department of General Surgery, Wafangdian Central Hospital, Dalian, Liaoning, China

**Keywords:** pancreatic cancer, oxidative stress, mitochondrial dysfunction, molecular heterogeneity, precision medicine, pancreatic neuroendocrine tumor

## Abstract

**Background:**

Mitochondria are significant both for cellular energy production and reactive oxygen/nitrogen species formation. However, the significant functions of mitochondrial genes related to oxidative stress (MTGs-OS) in pancreatic cancer (PC) and pancreatic neuroendocrine tumor (PNET) are yet to be investigated integrally. Therefore, in pan-cancer, particularly PC and PNET, a thorough assessment of the MTGs-OS is required.

**Methods:**

Expression patterns, prognostic significance, mutation data, methylation rates, and pathway-regulation interactions were studied to comprehensively elucidate the involvement of MTGs-OS in pan-cancer. Next, we separated the 930 PC and 226 PNET patients into 3 clusters according to MTGs-OS expression and MTGs-OS scores. LASSO regression analysis was utilized to construct a novel prognostic model for PC. qRT-PCR(Quantitative real-time PCR) experiments were performed to verify the expression levels of model genes.

**Results:**

The subtype associated with the poorest prognosis and lowerest MTGs-OS scores was Cluster 3, which could demonstrate the vital function of MTGs-OS for the pathophysiological processes of PC. The three clusters displayed distinct variations in the expression of conventional cancer-associated genes and the infiltration of immune cells. Similar molecular heterogeneity was observed in patients with PNET. PNET patients with S1 and S2 subtypes also showed distinct MTGs-OS scores. Given the important function of MTGs-OS in PC, a novel and robust MTGs-related prognostic signature (MTGs-RPS) was established and identified for predicting clinical outcomes for PC accurately. Patients with PC were separated into the training, internal validation, and external validation datasets at random; the expression profile of MTGs-OS was used to classify patients into high-risk (poor prognosis) or low-risk (good prognosis) categories. The variations in the tumor immune microenvironment may account for the better prognoses observed in high-risk individuals relative to low-risk ones.

**Conclusions:**

Overall, our study for the first time identified and validated eleven MTGs-OS remarkably linked to the progression of PC and PNET, and elaborated the biological function and prognostic value of MTGs-OS. Most importantly, we established a novel protocol for the prognostic evaluation and individualized treatment for patients with PC.

## 1 Introduction

Pancreatic cancer (PC) is one of the most prevalent types of malignancies that may develop in the digestive system and is the major contributor to cancer-associated deaths globally. Pancreatic neuroendocrine tumor (PNET) is a heterogeneous tumor originating from neuroendocrine system pluripotent stem cells. In the last 25 years, the cases of PC have increased by more than doubled (1). At this time, the incorporation of major surgery during the early stages of the disease is the most successful therapy for people who have PC and PNET. Over 80% of patients could not be identified at an early phase since there was a lacking of prominent early diagnostic signs and biological markers. During diagnosis, the majority of patients with PC are already in the terminal phase with distant metastases, which results in a loss of ideal prospects for surgery (2, 3). Furthermore, postoperatively, around 80% of individuals with PC often develop metastasis and experience disease recurrence (4). Notwithstanding the significant advancements in treatment methods, people diagnosed with PC continue to have a dismal prognosis worldwide (5). Hence, chemotherapy sensitivity for PC is still a challenge that has to be overcome. It is urgent warrant to establish an innovative and precise model for prognosis prediction in PC and PNET individuals.

Mitochondria acted as metabolic hubs at the cellular and tissue level, which were significant both for cellular energy production and reactive oxygen/nitrogen species (ROS/RNS) formation (6). Mitochondrial dysfunction (MDF) could produce mitochondrial reactive oxygen species (mtROS), which performs an instrumental function in diabetes mellitus, cancer, neurodegenerative diseases, and hydrocephaly (6, 7). The mtROS could activate signaling cascades that modify the expression of genes and influence cell activation, proliferation, and differentiation (8). Oxidative stress (OS) is an imbalance between the production of ROS/RNS and antioxidant protection effect, which is considered to be involved in varieties of disorders including DNA damage and repair (DDR) related diseases and neoplasm (9). Notably, the mitochondrial respiratory chain is the primary contributor to intracellular ROS and performs a fundamental function in modulating cellular redox homeostasis (10). Therefore, MDF could disrupt the normal functional activities of cells by increasing intracellular ROS (11). An overabundance of ROS will promote the oxidation of protein DNA and lipid macromolecules, resulting in genomic instability and further promoting transformation. Consequently, elevating ROS might promote the occurrence of tumors. Nevertheless, some research reports suggest increasing oxidative damage and enhancing ROS-dependent death signals might also be effective in preventing certain steps of tumorigenesis (12–14). Although oxidative stress-mediated alterations in mitochondria contribute significantly to tumorigenesis, their causative relationship is still up for debate (14). In addition, research has shown the molecular features of mitochondrial genes related to oxidative stress (MTGs-OS) and reported their predictive function in clear cell renal cell carcinoma (15). Based on the above analysis, we included MTGs-OS in this research. Moreover, no correlation between MTGs-OS and clinical outcomes or chemotherapy response has been reported in PC. Hence, the research exploring the expression patterns of MTGs-OS and constructing MTGs-OS molecular subtypes is promising.

In the study, PC patients’ prognoses were closely correlated with 56 MTGs-OS that were confirmed to be substantially upregulated in the disease. Next, we conducted a thorough analysis of the MTGs-OS expression profiles and genetic alterations across various cancers. We grouped PC and PNET patients into three clusters as per MTGs-OS scores and MTGs-OS expression and evaluated the correlations between these clusters and prognosis, immune microenvironments, and drug sensitivities. Furthermore, we developed and extensively validated a new and independent prognostic model using MTGs-OS. The approach has the potential to shed light on the mechanisms behind PC and PNET, leading to more targeted therapy and better clinical results for PC and PNET patients.

## 2 Methods

### 2.1 Data collection and processing

We downloaded the transcriptomic and corresponding clinical data of PC patients from the following four omics-related platforms: The Cancer Genome Atlas (TCGA, https://portal.gdc.cancer.gov/), ArrayExpress, Gene Expression Omnibus (GEO, https://www.ncbi.nlm.nih.gov/geo/), and International Cancer Genome Consortium (ICGC, https://dcc.icgc.org/) databases. In this analysis, only PC patients who had full follow-up data were included. Data from RNA seq (values expressed as FPKM) were translated into TPM for the TCGA-PC cohort to make them more similar to microarray results. To avoid the batch effect, we use the ComBat function in the SVA package to correct the data from different platforms (41). Overall, we complied the expression profile and clinical information of 930 PC patients consisting of TCGA-PC, GSE62452, GSE28735, GSE57495, ICGC-AU, ICGC-CA, and MTAB-6134 datasets (16–19).

We also downloaded and complied the transcriptomic data of PNET patients from the following three cohorts: GSE73338, GSE98894, and ICGC-PAEN-AU cohorts (20). Similar de-batch technologies were also employed to correct the data from different platforms. Overall, a total of 226 patients with PNET were obtained for the subsequent analysis.

Subsequently, 1136 MTGs were obtained from the MitoCarta3.0 database (http://www.broadinstitute.org/mitocarta), and using the query “oxidative stress,” the GeneCard database (https://www.genecards.org/) provided information on 9512 human genes associated with OS. Finally, 469 MTGs-OS were preserved for further analysis after taking the intersection.

### 2.2 Identification and validation of differentially expressed MTGs-OS associated with PC prognosis

Overall, 178 PC and 171 normal pancreas tissues in the GTEx and TCGA cohorts were obtained for performing differential expression analysis. Subsequently, prognosis-related analysis was conducted on 930 PC samples with complete follow-up records. The ‘limma’ tool in R was employed to evaluate differentially expressed MTGs-OS between PC and normal pancreas tissues from the GTEx and TCGA cohorts. To ascertain the predictive capabilities of MTGs-OS, we applied the “survival” and “survminer” R packages. After the intersection, clustering analyses and model development were carried out using the obtained differentially expressed MTGs-OS with predictive significance.

### 2.3 Pan-cancer analysis

To summarize the molecular traits of the above MTGs-OS in a variety of human cancers, pan-cancer analysis integrating genomics, transcriptomics, and clinical information was subsequently carried out (21, 22). First, we downloaded and compiled the raw data and clinical records of pan-cancer cohorts from the XENA website. We systematically studied whether the differentially expressed genes in PC and para-cancerous tissues had similar expression characteristics in other malignant tumors. In addition, we investigated the prognostic performances, mutation traits, and methylation levels of MTGs-OS in a series of human cancers. Finally, the ssGSEA algorithm was utilized to derive the MTGs scores of each cancer patient. The GSEA method was then applied to predict the discrepancies in pathway activities between patients with high- and low- MTGs scores (23).

### 2.4 Clustering analysis for 930 patients with PC

#### 2.4.1 MTGs scores classification of 930 PC patients into three clusters with significantly different prognoses and chemosensitivities

GSVA(Gene Set Variation Analysis) package was used to evaluate the MTGs scores of 930 patients with PC, which could indirectly reflect the relative activity of MTGs. A higher MTGs scores means that patients with a subtype of PC have a relatively high-activity MTGs phenotype, whereas a lower MTGs score implies a low-activity MTGs phenotype. Based on the MTGs scores, we then classified 930 patients with pancreatic cancer into three subtypes: MTGs active (cluster 2 or C2), MTGs inactive (cluster 3 or C3), and normal MTGs (cluster 1 or C1). Finally, the clustering outcome and the expression characteristics of each MTGs is shown in the form of a heat map.

To highlight the clinical value of MTGs scores, we compared the clinical outcomes and treatment strategies of different subgroups of pancreatic cancer patients. To examine the variation in prognosis among these 3 categories, the “survival” and “survminer” R programs were used. To derive the half-maximal inhibitory concentration (IC50) of the pharmaceuticals in each of the 3 clusters the “pRRophetic” program in R was applied. Notably, higher drug responsiveness was indicated by a lower IC50 value.

#### 2.4.2 Distributions of classical cancer-related genes and pathways across three clusters

The “string,” “pheatmap,” “gplots,” and “gird,” packages in R were employed to investigate the expression patterns of several tumor suppressor genes and oncogenes across the three clusters to gain insight into the possible regulation mechanism of the MTGs in PC. The expression profiles of numerous tumor suppressor genes and oncogenes in PC were subjected to comparison across clusters by means of the Kruskal-Wallis test. Furthermore, the enrichment scores of established metabolic and immune-related pathways were derived through ssGSEA, and a similar strategy was applied to characterize the differences in these pathways across three MTGs clusters.

#### 2.4.3 Association of MTGs scores with immune cell infiltration

Similar to the method previously reported in the literature (24), the ssGSEA algorithm was adopted to evaluate the characteristics of the immune microenvironment, including immune cell infiltrations and immune-related functions, in 930 patients with PC. To further investigate the critical modulatory role of MTGs on the immune microenvironment in PC, we assessed the association of each MTG with the aforementioned immune cell infiltrations and immune-related activities using the Spearman test. We computed the spearman’s correlation value and produced a lollipop map to illustrate our findings on the association of MTGs score with immune cell infiltration in PC from a wider perspective. In addition, we generated a scatter plot with the “ggscatterstats” package to display the associations of the five classical immune traits (neutrophils, APC co-inhibition, CCR, T cell co-inhibition, and macrophages) with the MTGs scores.

More importantly, a series of immune-prediction algorithms were applied to intensively evaluate the discrepancies in the immune cell infiltration among different subpopulations of PC patients. First, based on the transcriptomic data, the “ESTIMATE” package was adopted to evaluate the variations in immunological features across the three PC subtypes (on the basis of the Immune Score, Stromal Score, and ESTIMATE Score) (25). Then, cell immune responses or cellular components were contrasted among the three PC subtypes using the EPIC, MCPCOUNTER, TIMER, XCELL, CIBERSORT-ABS, and CIBERSORT, algorithms (26). We conducted the Kruskal-Wallis test to examine the relative differences in immune checkpoint expression across distinct subtypes of PC, as immune checkpoints perform a fundamental role in the functioning of immune cells.

### 2.5 A novel prognostic signature development and verification

#### 2.5.1 Development and verification of a novel MTGs-related prognostic signature for 930 PC patients

The expression of MTGs-OS was used to develop an innovative prognostic signature that was verified to more accurately predict a patient’s chance of survival. Signature cohort 1 comprised 635 patients from the TCGA-PC, MTAB-6134, GSE62452, GSE57495, and GSE28735 datasets, whereas signature cohort 2 comprised 295 patients from the ICGC-CA and ICGC-AU datasets.

Subsequently, approximately 70% of samples in signature cohort1 were randomly obtained and designated as the train dataset used for developing MTGs-RPS (n=447). The remaining 30% of samples in signature cohort1 were defined as the test1 cohort (n=188). All samples in signature cohort1 were allocated to the test2 cohort (n=635) and all samples in the signature cohort2 were allocated to the test3 cohort (n=295).

To avoid overfitting and identify feasible factors from the MTGs, a least absolute shrinkage and selection operator (LASSO) regression analysis was conducted on the train set. Subsequently, to develop an MTG-RPS, a multivariate Cox proportional hazards regression model was established, and the risk scores were estimated with the help of the equation below: risk score = $\sum_{k=1}^{n}expk*\beta k$ . Following the completion of the calculations necessary to determine each patient’s individual risk score within the train set, patients were then classified into high-risk and low-risk groups (categories) as per the median risk score. The PC patients in the internal (i.e. test1 and test2) and external (test3) validation datasets were further classified into high- and low-risk categories as per the train cohort-related median risk score.

The analyses below were conducted on the train, (i.e. test1 and test2) and external (test3) validation datasets to develop and validate the MTGs-RPS: (1) The ‘pheatmap’ R program was utilized to produce a heatmap that illustrates the various degrees of expression of the MTGs. (2) A survival study was conducted with the Kaplan-Meier (KM) method to ascertain if the MTGs-RPS is useful for forecasting prognosis; (3) We created receiver-operating characteristic (ROC) curves for evaluating the diagnostic capability of the MTGs-RPS based on the area under the curve (AUC).

#### 2.5.2 The validation of the hub RNA expression with quantitative real-time PCR

The cell lines H6C7(Human pancreatic ductal epithelial cells, HPDE6-C7), and the PC cell lines CF-PAC1, BxPC-3, and Panc-1 were supplied by the ATCC company. The DMEM augmented with 10% FBS (Gibco, USA) was used to culture H6C7 and Panc-1 cell lines. Next, the BxPC-3 cell line was cultured in a 1640 medium with 10% FBS (Gibco, USA). Additionally, the CF-PAC1 cell line was cultured with IMDM with 10% (FBS) (Procell, China).

The total mRNAs of H6C7, CF-PAC1, Panc-1and BxPC-3 cell lines were extracted by the TRIzol kit. The cDNAs of H6C7, CF-PAC1, Panc-1and BxPC-3 cell lines were obtained by the reverse transcription of the mRNAs using the Reverse Transcription Reagent. The qPCR Kit (Accurate Biotechnology) was employed to conduct RT-PCR. GAPDH served as the control standard. Thereafter, the level of RNA expression was analyzed and quantified by the ΔΔCt method. Below are the sequences of the primers employed to investigate the 11 human hub genes: COQ4, 5’- CCTGTCCTCCGTCGGCTCTG-3’ (Forward), 5’- GTGGGGAGGTGGTGCGAGTATAG-3’ (Reverse); MCRIP2, 5’- ATGTCCGCTTTGTGTCCGAAGAC-3’ (Forward), 5’- GGTGATTCTCGCCAGGAACTGC-3’ (Reverse); SOD1, 5’- TGTTGGAGACTTGGGCAATGTGAC-3’ (Forward), 5’- ACCAGTGTGCGGCCAATGATG-3’ (Reverse); NDUFB8, 5’- ACAACAGGAACCGTGTGGATACATC-3’ (Forward), 5’- TGAAAGCCAGGAAACCGAAGAGC-3’ (Reverse); MRPL50, 5’- AATTGGCAAGACATCTCCCTGGAAG-3’ (Forward), 5’- ACATCTGGTGGAGTCTGGAGTTAGG-3’ (Reverse); BIK, 5’- GAGGGCAGTGACGCATTGGC-3’ (Forward), 5’- CCTCAGTCTGGTCGTAGATGAAAGC-3’ (Reverse); MRPL14, 5’- AGCCATCACTGTTTCAGCACCAC-3’ (Forward), 5’- GCACTGTTGTCCACCACTCGTAC-3’ (Reverse); RFK, 5’- GATGGTGGTGAGCATAGGATGGAAC-3’ (Forward), 5’- TTCTGGTCTCAGGTAGCCAACAATG-3’ (Reverse); NMNAT3, 5’- CAGCAAGACACCATCAGCCTCTG-3’ (Forward), 5’- ACGCACACCAAGCCGAACTTC-3’ (Reverse); BNIP3L, 5’- CCATCCTCATCCTCCATCCACAATG-3’ (Forward), 5’- CGAAGGGCTGTCACAGTGAGAAC-3’ (Reverse); MRPS5, 5’- GATCCGTGTCTTGGTGGCTGTG-3’ (Forward), 5’- TGAAAGCATCCATCCGATCAGTAGC-3’ (Reverse).

#### 2.5.3 The discrepancy of drug sensitivity, immune cell infiltration, and immune checkpoint genes expression in low- and high-risk subgroups

Drug sensitivity was predicted for each PC patient using the “pRRophetic” R package, and possible sensitive medicines were screened using the “Wilcox.test” R function. The only medications that were regarded to be effective targeted treatments were those that showed statistical significance across all four groups (train, test1, test2, and test3). Notably, higher drug sensitivity corresponds to a low IC50 value.

Cell immune responses or cellular components were evaluated using the CIBERSORT-ABS, XCELL, EPIC, MCPCOUNTER, CIBERSORT, and TIMER algorithms and compared between model-based low- and high-risk categories. Additionally, we explored if there were any variances in ICG expression among low- and high-risk patients. Only the ICGs with significant statistical differences were displayed.

### 2.6 Clustering analysis for 226 patients with PNET

The intrinsic molecular heterogeneity of patients with PNET was identified by a method similar to that of previous cluster analysis. Firstly, the MTGs scores of each PNET patient was evaluated based on the GSVA package, and the MTGs scores were combined with the expression profile of related genes. Based on the MTGs scores and gene expression profile, cluster analysis was carried out in patients with PNET. Similarly, 226 patients with PNET were divided into C2 and C3 subtypes, and the MTGs scores of different subtypes were compared. The two subtypes with no difference in MTGs scores were further merged to get S1 and S2 PNET genotypes, and the MTGs scores between S1 and S2 were compared again. The classical metabolic pathway score and immune pathway score of each PNET patient were calculated based on GSVA package to characterize the inherent molecular characteristics of different PNET subtypes.

## 3 Results

### 3.1 Detection of differentially expressed MTGs linked to PC prognosis

**Figure 1:**
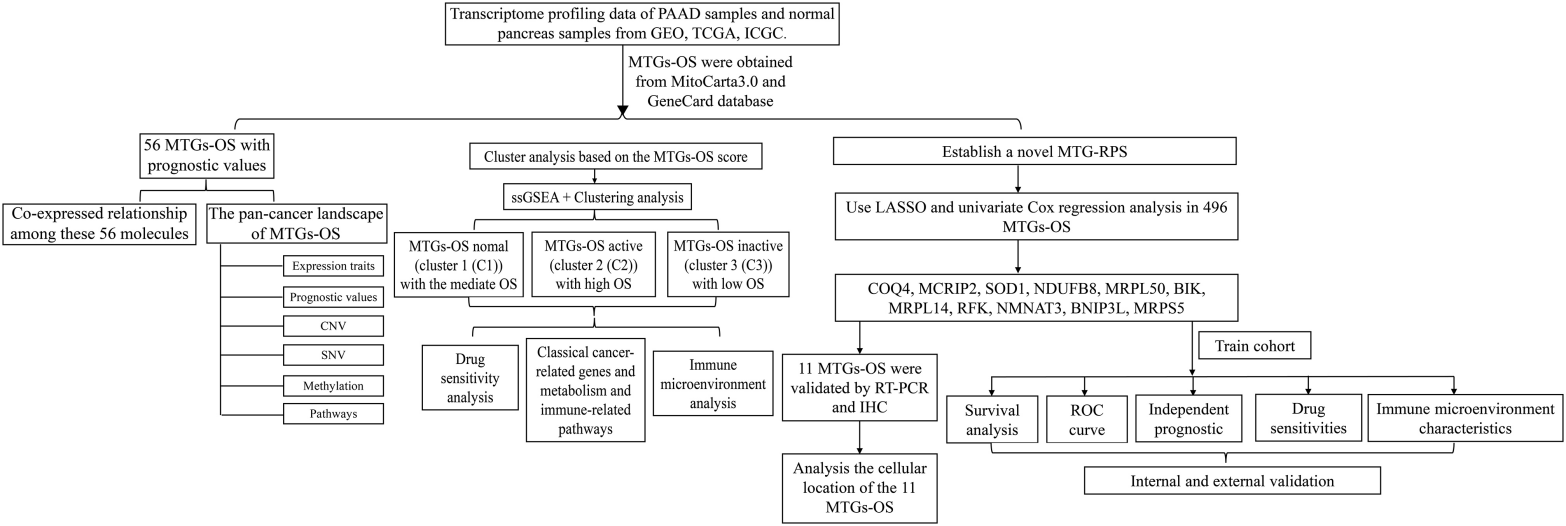
The detailed analytic workflow of the research.

**Figure 2:**
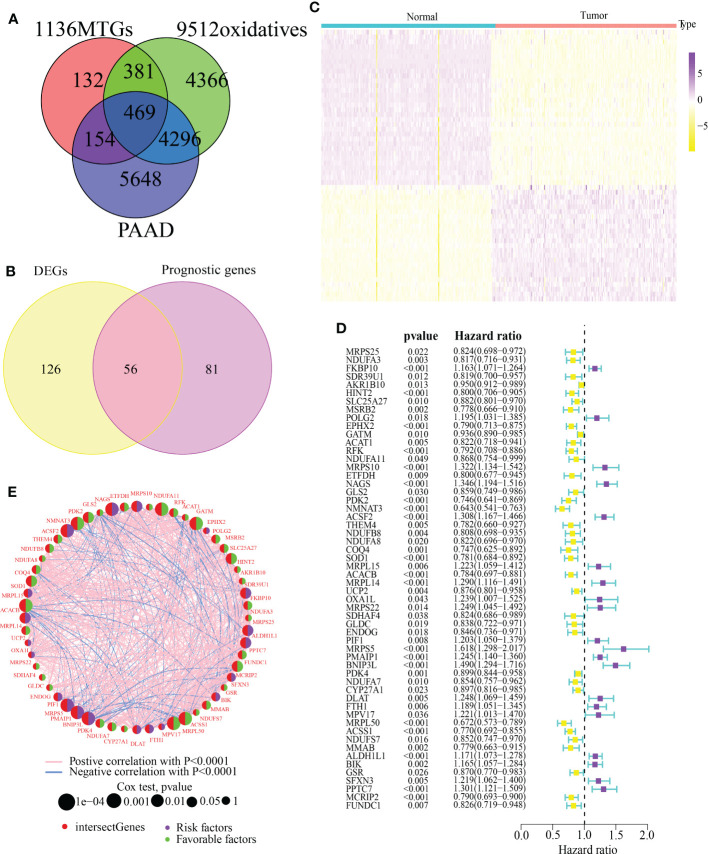
Determining MTGs with differential expression and their prognostic value. **(A)** A Venn diagram revealed 469 MTGs associated with oxidative stress that were involved in PAAD. **(B)** 56 differentially expressed MTGs-OS with prognostic values were identified. **(C)** A Heatmap depicted above 56 candidate genes with differential expression between PAAD and normal pancreatic tissues. **(D)** A prognosis-related forest depicted above 56 candidate genes. **(E)** The co-expressed relationship of 56 candidate genes.

The detailed analytic workflow of this research was displayed in **Figure 1**. Through the classical bioinformatics difference analysis, we found 469 MTGs-OS in PC (**Figure 2A**). Among 469 MTGs-OS, 182 differentially expressed MTGs-OS were discovered using the lima program in R (**Supplementary Table 1**). More importantly, 137 MTGs-OS were detected to be remarkably associated with PC prognoses (**Supplementary Table 2**). After the intersection, 56 differentially expressed MTGs-OS with prognostic values were obtained (**Figure 2B**). The differential expression profiles of above 56 candidate genes were demonstrated in **Figure 2C**. The prognostic forest plot was also showed in **Figure 2D**. Notably, most MTGs-OS showed obvious down-regulated expression tendency in PC tissues and were closely associated with favorable prognoses, suggesting their protective roles of MTGs-OS in PC. In addition, the co-expressed relationship among these 56 molecules was intensively explored (**Figure 2E**).

### 3.2 Pan-cancer characterization of 56 differentially expressed MTGs-OS with prognostic values

**Figure 3:**
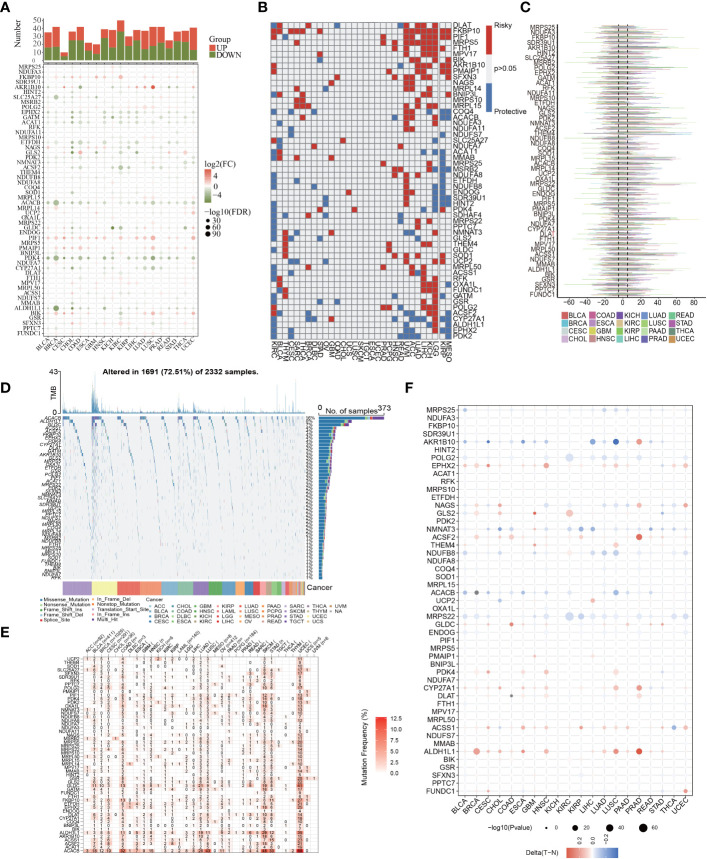
Pan-cancer characterization of 56 differentially expressed MTGs-OS. **(A)** The expression characteristics of 56 MTGs-OS in other malignant tumors (P<0.05). **(B)** Survival profiles of the MTGs-OS pan-cancer. Genes with P > 0.05 are highlighted in white, whereas the risk and protective genes are denoted by the red and blue colors, correspondingly. **(C)** Gain and loss frequencies of CNVs in the 56 MTGs-OS in various cancers. The frequency variation of MTGs-OS in pan-cancers is shown by the line’s length. **(D, E)** SNV data of the 56 MTGs-OS depicted in pan-cancers by heatmap and waterfall plot. **(F)** The DNA methylation of 56 MTGs-OS in pan-cancers (the gradient from red to blue represents a decline from a high to a low level).

Then, to examine the molecular traits of the 56 MTGs-OS in other human cancers, pan-cancer analyses were carried out based on genomics, transcriptomics, and clinical information. First, we researched the expression characteristics of 56 MTGs-OS in other malignant tumors (**Figure 3A**). Most of the 56 MTGs-OS were expressed differentially between tumors and adjacent normal samples in diverse cancers, including CHOL, COAD, KIRC, UCEC, and LUSC. Besides, the levels of FKBP10, PIF1, and PMAIP1 in nearly all tumor tissues were elevated in contrast with those in paired normal samples; conversely, in almost every case, tumor tissues had lower expression of PDK4, ALDH1L1, ACACB, ACSF2, EPHX2, GATM, ACAT1, and ETFDH than their matched normal tissues. The association of gene expression with patient survival time was then evaluated, and the predictive capabilities of the 56 MTGs-OS in pan-cancers were investigated. As depicted in **Figure 3B**, we performed a univariate cox regression analysis to determine the MTGs-OS that served as risk factors (HR>1 and p<0.05) and those with protective function (HR<1 and p<0.05). We discovered that certain MTGs-OS are risk factors in part of malignancies, including UVM, ACC, LIHC, KICH, and LGG; however, similar to the protective function of MTGs-OS in PC, these genes also had protective properties in KIRC, MESO, and KIRP. To investigate the mutational characteristics of the 56 MTGs-OS in tumors, the proportion of CNV was studied, and the outcomes showed that CNV happened at high frequency (nearly within the range of 10 to 60%) in the diverse types of cancer (**Figure 3C**). Then, we discovered that BLCA, BRCA, COAD, LUAD, SKCM, STAD, and UCEC, all exhibited a higher frequency of SNVs, especially for UCEC; conversely, the SNV frequency in CHOL, ACC, DLBC, UVM, THYM, THCA, TGCT, PCPG, MESO, LAML, and KICH was low, especially for CHOL and UVM. Interestingly, we found that the SNV of ACACB, ALDH1L1, and GLDC was in significantly high frequencies than that of the other MTGs-OS, as illustrated by the heatmap and waterfall diagram of SNV (**Figures 3D, E**). Notably, the methylation of promoters and abnormal DNA methylation might influence gene expression (27). Therefore, we discovered that MTGs-OS had complicated methylation patterns in different types of cancer. As a whole, the changes in methylation levels of MTGs-OS were not significant in most human cancers, including PC, according to the result (**Figure 3F**). EPHX2, ACSF2, CYP27A1, ACSS1, and ALDH1L1 showed hypermethylation in several types of human cancer; on the contrary, AKR1B10, POLG2, NDUFB8, ACACB, and MRPS22 consistently showed hypomethylation in several types of human cancer.

### 3.3 Cluster analysis for 930 PC patients based on MTGs-OS scores

**Figure 4:**
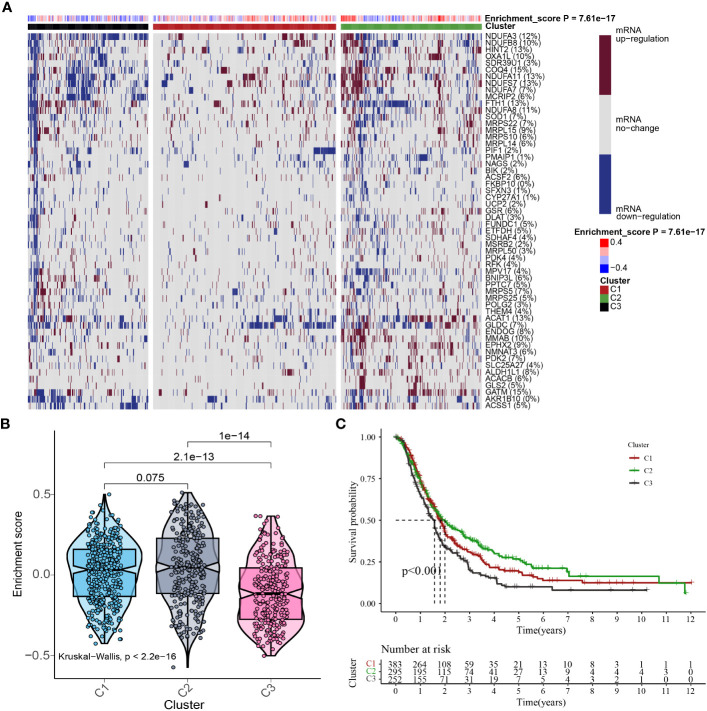
Cluster analysis based on MTGs-OS scores. **(A)** In this case, the heat map shows three distinct clusters based on the gene data: Clusters C1, C2, and C3, as per MTGs-OS mRNA expression levels. The tumor patients with normal, active, and inactive MTGs-OS were categorized under C1, C2, and C3, respectively. The right-hand bar’s colors indicate the degree of mRNA regulation: white for no regulation, blue for downregulation, and red for upregulation. The MTGs-OS is presented in four distinct hues, with blue signifying negative values and red signifying positive ones. **(B)** As depicted in the violin plot, the enrichment scores for each of the three clusters were ranked as follows: C2 > C1 > C3. On top of each cluster, the corresponding p-values are shown. **(C)** The three clusters are represented by a survival curve. In comparison to C1 and C2, cluster 3 has a lower chance of survival. Red, green, and black lines denote C1, C2, and C3, respectively. The x-coordinate is survival time, while the ordinate is the survival rate.

In consideration of the function and impact of MTGs-OS in PC, the enrichment scores of MTGs-OS were initially evaluated *via* ssGSEA for a sum of 930 patients. The 930 different PC samples were divided up into three unique categories (Cluster1, Cluster C2, and Cluster C3) as per the concentrations of mRNA that MTGs-OS expresses (**Figure 4A**). C1, C2, and C3 comprised patients with normal, active, and inactive MTGs-OS, respectively. The violin plot illustrates that C2 has the highest enrichment score out of the three clusters, followed by C1 and C3 (**Figure 4B**). Subsequently, by plotting the survival curves for the three categories, we assessed the reliability of the clustering technique. As for the 3 clusters, we found that patients in C3 exhibited the lowest overall survival rates whereas those in C2 exhibited the highest overall survival rates (**Figure 4C**), which revealed that the MTGs-OS score was a protective indicator. The result in the part was in accordance with the results of previous univariate Cox regression analysis. Overall, based on these findings, we conclude that our PC classification technique is accurate, credible, and scientific.

### 3.4 Link between drug sensitivity and MTGs-OS scores

**Figure 5:**
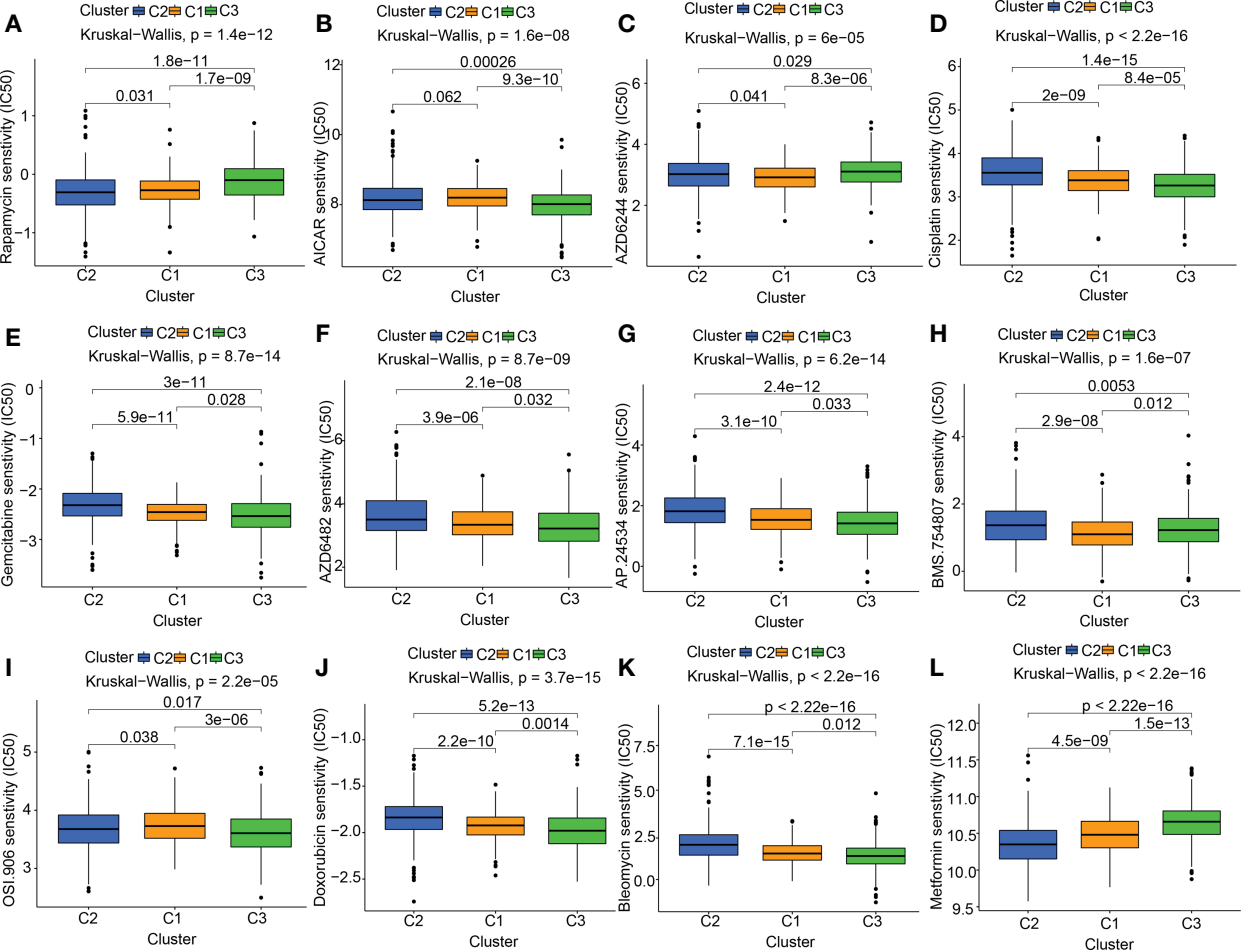
The link between drug sensitivity and MTGs-OS scores. The box plot for the estimated IC50 of 12 different types of commonly used chemotherapy regimens conducted in **(A–L)** for C1 (yellow), C2 (blue), and C3 (green). The 12 different classes of chemotherapy regimens include Rapamycin, AICAR, AZD6244 (selumetinib), cisplatin, gemcitabine, AZD6482 (PI3Kβ inhibitors), AP.24534 (ponatinib), BMS.754807 (IGF-1R/IR inhibitor), OSI.906 (linsitinib), doxorubicin, bleomycin, and metformin. On top of each cluster, the corresponding p-values are shown, and the P< 0.05 denotes the significance level.

At present, the treatment status of pancreatic cancer is still grim. The new concept of oncology has been instrumental in the rapid advancement of cancer detection and therapy in recent years. Targeted therapy and immunotherapy have advanced the comprehensive diagnostic testing and management of PC beyond the reach of standard therapies like surgery, chemotherapy, and radiation treatments. The foundation of molecular targeted therapy is the knowledge of the molecular biology of neoplasm. Key molecules associated with cancer were identified, which might be used as therapeutic targets. According to the characteristics of the therapeutic targets, using highly precise medications and agents to treat cancer is promising. Based on previous research reports, mitochondrial-related OS participates in the onset and progression of tumors. We also discovered that the MTGs-OS score was a marker that might be protective for those with PC. Therefore, we evaluated the connection between MTGs-OS and the effectiveness of targeted pharmaceuticals already available for treating PC. Twelve drugs were chosen, including mTOR inhibitor (Rapamycin), AMPK activator (AICAR), MEK inhibitors (Selumetinib) plant anticancer drugs (cisplatin), pyrimidine antineoplastic drugs (gemcitabine), PI3Kβ inhibitors (AZD6482), tyrosine kinase inhibitor (ponatinib), IGF-1R/IR inhibitor (BMS.754807, linsitinib), metformin and the other chemotherapeutic drugs (doxorubicin, bleomycin). We applied the “pRRophetic” program to make a comparison of the IC50 values of the 12 drugs across these three molecular subgroups. We compiled a list of MTGs-OS clusters and their associated medication sensitivities: Rapamycin: C2 > C1 > C3; AICAR: C3 > C2 > C1; AZD6244(selumetinib): C1 > C2 > C3; cisplatin: C3> C1 > C2; gemcitabine: C3> C1 > C2; AZD6482(PI3Kβ inhibitors): C3> C1 > C2; AP.24534 (ponatinib): C3> C1 > C2; BMS.754807 (IGF-1R/IR inhibitor): C1> C3 > C2; OSI.906(linsitinib): C3 > C2 > C1; doxorubicin: C3> C1 > C2; bleomycin: C3>C1>C2 (**Figures 5A–L**).

### 3.5 Correlation of MTGs-OS scores with potentially targetable classical genes, metabolism, and immune-related pathways

**Figure 6:**
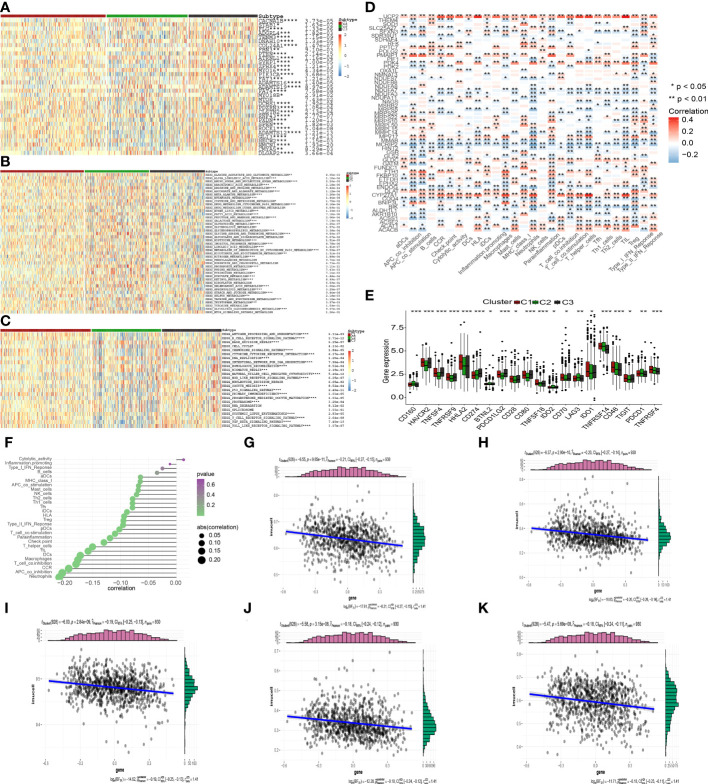
Correlation of MTGs-OS scores with classical oncogenes, metabolism, and immune-related pathways. **(A)** Three MTGs-OS clusters show varying degrees of expression of different oncogenes and tumor suppressor genes. **(B)** The activity of well-recognized metabolism in the 3 clusters. **(C)** The activity of well-recognized and immune-related pathways in the 3 clusters. **(D)** A link between MTGs-OS and immune cell infiltration is depicted *via* a heat map. Positive and negative associations are depicted in red and blue, respectively. **(E)** Immune checkpoint expression differs considerably across the three MTGs-OS clusters. **(F)** An illustration of the link between MTGs-OS and immune cell infiltration status. The dimension of the sphere of the figure represents the degree of abs (correlation), whereas the color represents the p-value. **(G–K)** The scatter plot draws the relationship of MTGs-OS with 5 immune-infiltration-related substances. The MTGs-OS have a negative link to the infiltration status of neutrophils, APC co-inhibition, CCR, T cell co-inhibition, and macrophages. (* denotes p<0.05; ** denotes p< 0.01; *** denotes p< 0.001;**** denotes p< 0.0001).

To examine the possible regulation mechanism of the MTGs-OS in PC, we evaluated the expression profiles of tumor suppressor genes and oncogenes in the 3 clusters, as illustrated by the heatmap in **Figure 6A**. We found that the expression levels of most of the oncogenes were much higher in C3 compared with C1 and C2, such as STAB1, TENM3, PIK3CA, ZFHX4, ADAMTS12, GLI3, HMCN1 and so on. On the contrary, C3 was shown to have a lower expression of tumor suppressor genes including RNF43, in contrast with C1 and C2. The stimulation of oncogenes and inhibition of suppressor genes probably resulted in the worst prognosis in C3. Interestingly, as was the case in the oncogenes, we discovered that the expression patterns of several tumor suppressor genes, such as PTEN, were highest in C3, but elevated in C1 and C2. We also examined the levels of activity in commonly recognized metabolic and immune-related pathways across the three clusters and computed the enrichment scores by ssGSEA (**Figures 6B, C**). Interestingly, various metabolism and immune-related pathways exhibited significant differences in the 3 clusters. We found that most of the protective metabolism pathways including KEGG_GLYCINE_SERINE_AND_THREONINE_METABOLISM were more inactive in C3 than that in C1 and C2. Also, almost all the immune-associated pathways were more active in C3 than in C1 and C2, such as KEGG_P53_SIGNALING_PATHWAY and KEGG_PROGESTERONE_MEDIATED_OOCYTE_MATURATION, which suggested that the degree of malignancy for PC was highest in the C3 leading to the worst prognosis. Additionally, the aforementioned findings established that MTGs-OS were protective factors for PC since they were strongly linked to metabolic reprogramming and immune-related pathways.

### 3.6 Correlations of the MTGs-OS scores with immune cell infiltration

**Figure 7:**
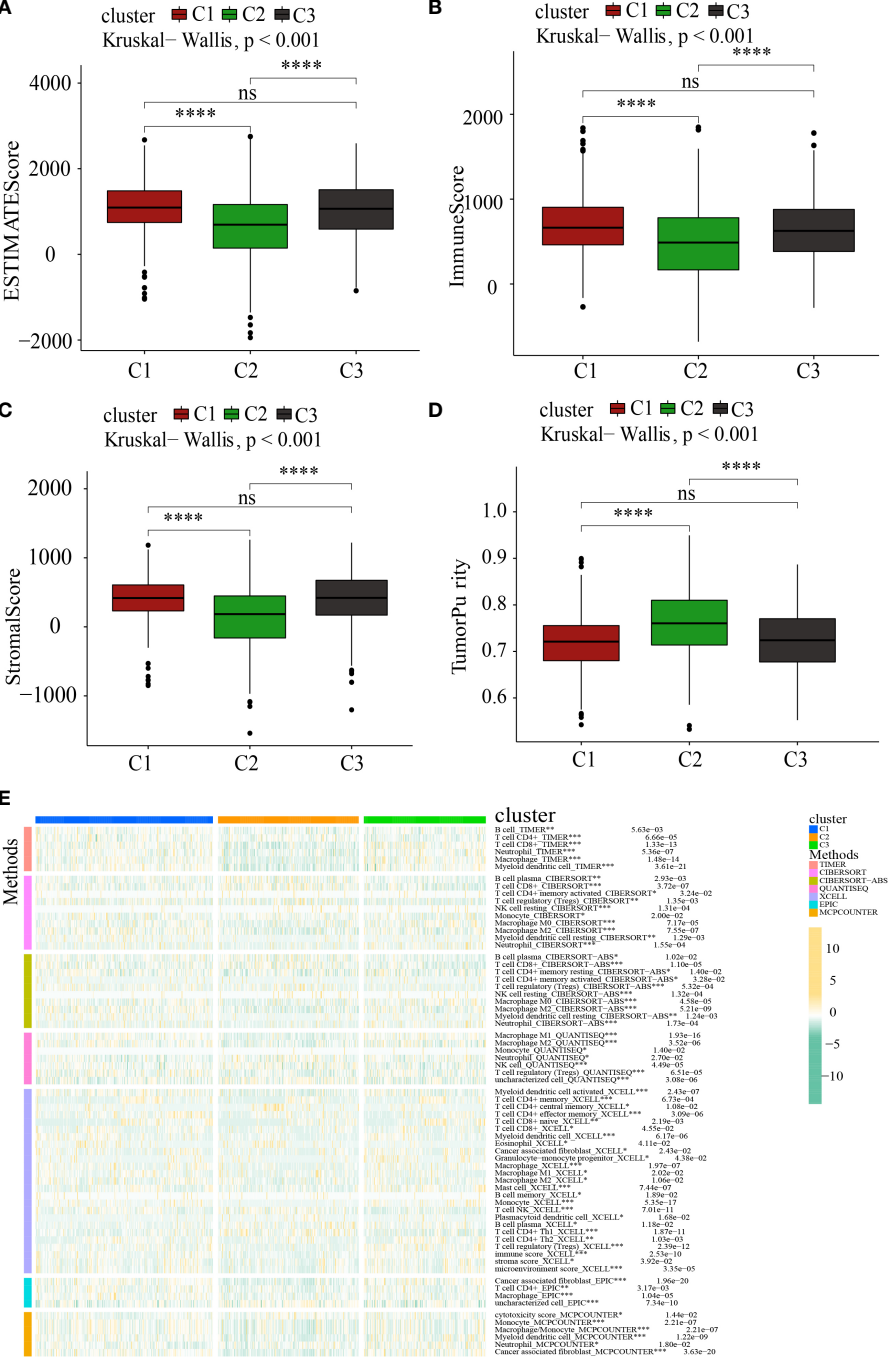
Systematic analysis of the tumor immune microenvironment and immune cell infiltration in the C1, C2, and C3 subgroups. **(A–D)** Comparison of the tumor immune microenvironment components in the C1, C2, and C3. **(E)** The distribution patterns of immune cell infiltration in the C1, C2, and C3 subsets. *P<0.05; **P<0.01; ***P<0.001; ****P <0.0001; ns, no significance.

It has been well-recognized that the tumor microenvironment (TME) performs an instrumental function in PC development (28, 29). It has been postulated that the TME, particularly the features of tumor-infiltrating immune cells (TIICs), might be used as treatment targets for cancer (30). The levels of ROS in the TME are critical for regulating the levels of immune cells and their functions in the TME, particularly the activation, expansion, and effector function of T lymphocytes (31, 32). Within the scope of this research, we delved deeper into the link between MTGs-OS and the infiltration levels of immune cells as well as the corresponding immune functions (**Figure 6D**). The expression of following genes, NDUFA7, NDUFA3, MMAB, MCRIP2, and COQ4, were negatively correlated with immunocyte infiltration and immune-related functions. Conversely, UCP2, ACAT1, CYP27A1, DLAT, FTH1, MPV17, PMAIP1, PPTC7, and GLDC, were positively linked to the infiltration levels of immune cells. Notably, ICGs contributed to anti-tumor immunity by modulating the function of effector T lymphocytes. Therefore, we studied the relationship between ICGs in the 3 clusters (**Figure 6E**). We discovered that the MTGs-OS-inactive cluster was correlated with overexpression of ICGs, illustrating that MTGs-OS-inactive patients had the weakest anti-tumor immunity and a poor prognosis. Subsequently, we assessed the association of MTGs-OS scores with immune cell infiltration (**Figures 6F–K**). Most infiltrating immune cells, comprising neutrophils, APC co-inhibition, CCR, T cell co-inhibition, and macrophages, were shown to have a negative link to MTGs-OS scores. The “ESTIMATE” R program was also used to examine the TIME of the three subtypes. The immune, stromal, and ESTIMATE scores, as well as the tumor purity, were depicted in **Figures 7A–D**. We discovered that C2 had considerably decreased stromal, immune, and ESTIMATE scores in contrast to C3. On the contrary, although the tumor purity in C2 was substantially increased in contrast with that in C3, the patients in C2 had a better prognosis. At the same time, we evaluated immune cell responses or cellular components in the three PC subtypes according to the EPIC, MCPCOUNTER, CIBERSORT-ABS, XCELL, CIBERSORT, and TIMER algorithms (**Figure 7E**). Overall, the cellular immune responses or cellular components were significantly different among three clusters, which suggested that there was a close link between the infiltration status of most immune cells and the MTGs-OS score.

### 3.7 Construction and verification of a novel MTGs-RPS for predicting the clinical outcomes of patients with PC

**Figure 8:**
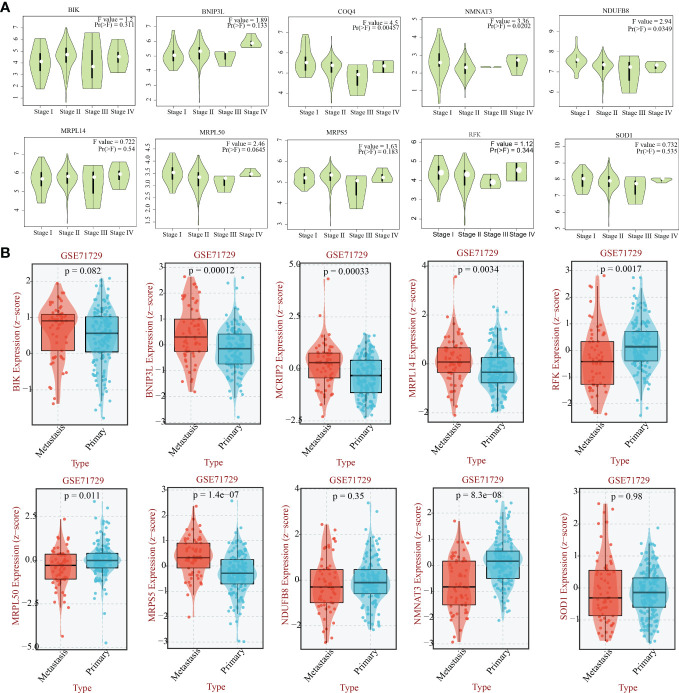
The relationship between the eleven genes and the stage and the metastasis capability of PAAD. **(A)** The relationship between 10 hub -genes(COQ4, SOD1, NDUFB8, MRPL50, BIK, MRPL14, RFK, NMNAT3, BNIP3L, MRPS5) and the stage of PAAD. **(B)** The relationship between 10 hub -genes(MCRIP2, SOD1, NDUFB8, MRPL50, BIK, MRPL14, RFK, NMNAT3, BNIP3L, MRPS5) and the metastasis capability of PAAD.

Previous 56 differentially expressed MTGs-OS with prognostic values were used for conducting the following LASSO-Cox regression analysis. LASSO regression analysis identified 18 candidate molecules and multivariate Cox regression analysis determined 11 hub MTGs-OS (i.e., COQ4, MCRIP2, SOD1, NDUFB8, MRPL50, BIK, MRPL14, RFK, NMNAT3, BNIP3L, MRPS5) for constructing the prognostic model (**Supplementary Figures 1A–C**). To additionally validate the fundamental function of the selected modeling genes in the onset and progression of PC, we investigated the association of the eleven model genes with PAAD stage and metastasis based on the GEPIA and BEST platforms (**Figure 8**).

**Figure 9:**
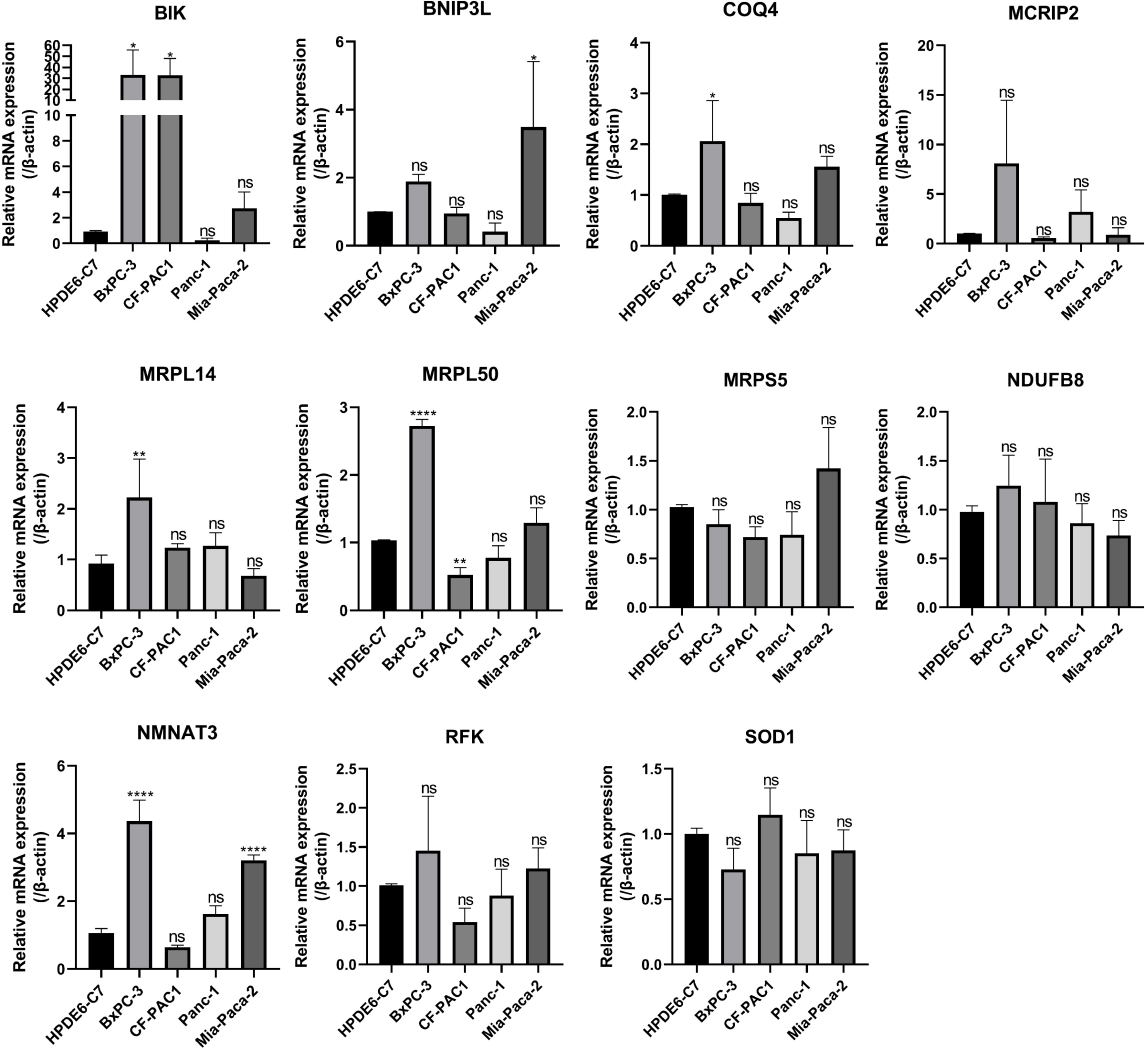
Verification of the expression levels of the eleven hub MTGs-OS by the RT-PCR. *P<0.05; **P<0.01; ****P<0.001; ns, no significance.

**Figure 10:**
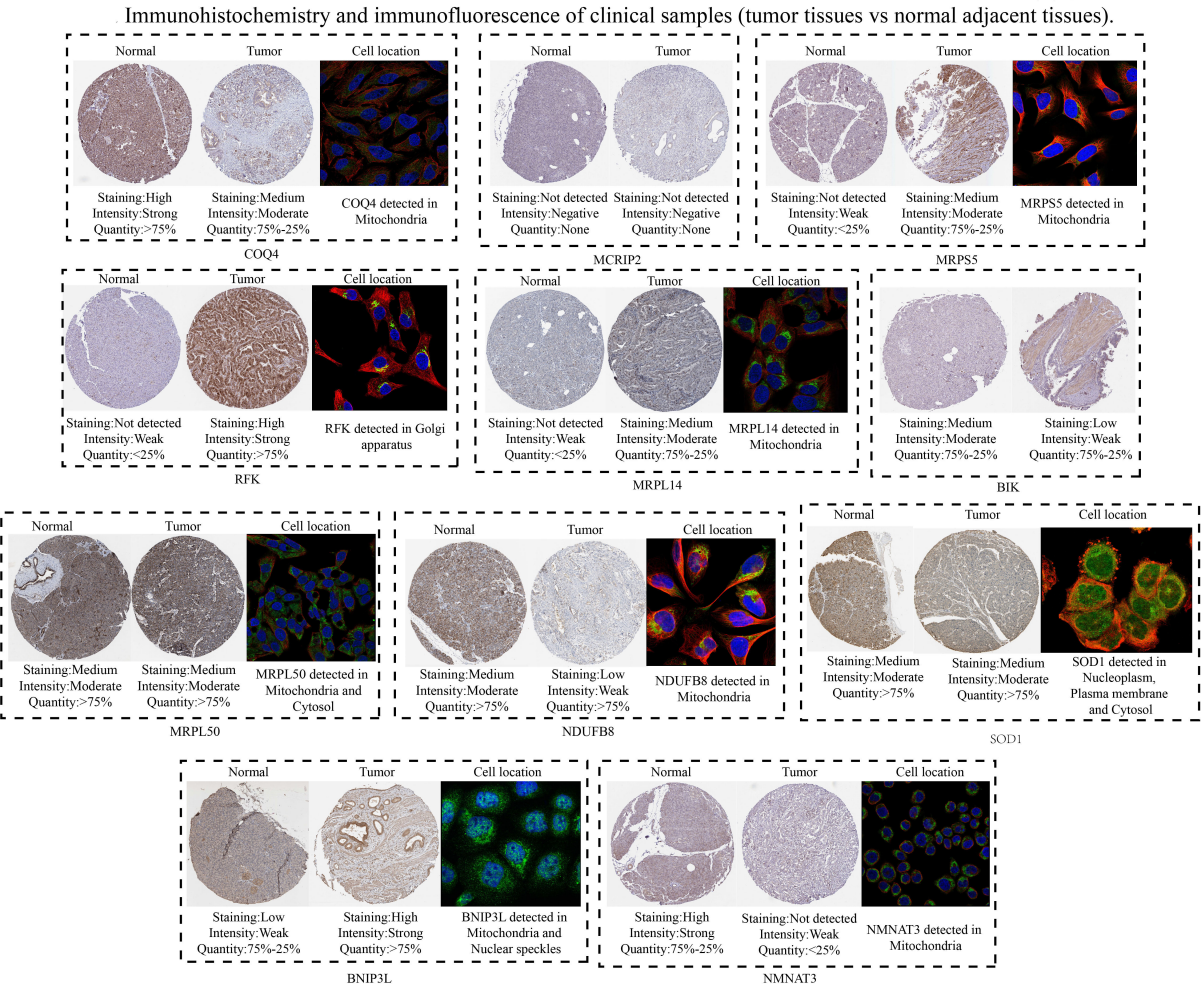
Verification of the expression levels of the eleven hub MTGs-OS by the immunohistochemical and immunofluorescence.

Subsequently, 11 hub MTGs-OS’s expression levels were verified by RT-PCR in four cell lines (**Figure 9**). We observed that the mRNA expression trend of the 11 hub MTGs-OS was almost consistent with the results predicted by our bioinformatics analysis. As for BxPC-3 cell line, the expression levels of BIK, COQ4, MRPL14, MRPL50, and NMNAT3 were significantly up-regulated. As for CF-PAC1 cell line, the expression level of BIK was significantly up-regulated and the expression level of MRPL50 was significantly down-regulated. As for Panc-1 cell line, there is no significant difference as regards above eleven model genes. As for Mia-Paca-2, the expression levels of BNIP3L and NMNAT3 were significantly up-regulated. Using the HPA database, we searched through immunohistochemical findings of tumor and normal pancreatic tissues to verify the expression pattern of 11 hub MTGs-OS at the protein levels (**Figure 10**). At the same time, we studied the cellular localization of the 11 MTGs-OS, however, we did not find the information of cellular localization of BIK and MCRIP2 in the HPA database. Therefore, we showed cellular localization of the other 9 MTGs-OS (COQ4, SOD1, NDUFB8, MRPL50, MRPL14, RFK, NMNAT3, BNIP3L, MRPS5) in **Figure 10**. The expression products of COQ4, MRPS5, MRPL14, NDUFB8, and NMNAT3 were mainly located in the mitochondria. The expression products of RFK, MRPL50, and SOD1 were located Golgi apparatus, mitochondria and cytosol, nucleoplasm and plasma membrane and cytosol, and mitochondria and nuclear speckles, respectively.

**Figure 11:**
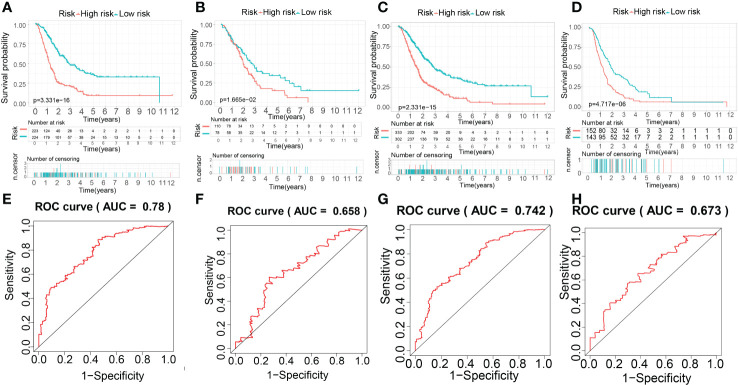
The establishment and evaluation of the prognostic performance of the MTG-RPS. **(A–D)** Survival curve of overall survival time between low- and high-risk patients of the MTG-RPS in the train, test1, test2, and test3 cohort. **(E–H)** ROC curve of 5 years between low- and high-risk patients of the MTG-RPS in the train, test1, test2, and test3 datasets with the area under the curve (AUC) of 0.780, 0.658, 0.742, and 0.673, respectively.

Based on the result above, the evaluation of the prognostic performance of the MTGs-RPS was displayed in **Figure 11**. PC patients in the train set were classified into two subgroups by KM survival analysis. The high-risk category recorded a lower overall survival time than the low-risk category, which suggested that the MTGs-RPS could precisely differentiate patients with a poor and a favorable prognosis for PC (**Figure 11A**). As per ROC analysis, we proved the accuracy and robustness of MTGs-RPS as a diagnostic tool. The 5-year survival AUC of the ROC curve AUC was 0.780 (**Figure 11E**). Furthermore, both internal and external validations of survival data found that PC patients with low-risk scores recorded superior overall survival rates (**Figures 11B–D**). In addition, when comparing the test1 with the test2 and external validation sets, we discovered that the AUC values of the 5-year ROC curve were 0.658, 0.742, and 0.673, respectively (**Figures 11F–H**). The results above could suggest the dependability and accuracy of the MTGs-RPS.

During the process of establishing the MTGs-RPS, we classified 447 PC patients into low- and high-risk categories as per the median risk in the train set (**Supplementary Figure 2A**). As per the distributions of risk scores, the mortality rate was remarkably greater among the high-risk subset of PC patients (**Supplementary Figure 2E**). The prognostic signature encompassed 11 different MTGs-OS, and their expression patterns were displayed in a heatmap (**Supplementary Figure 2I**). As we had anticipated, the same technique effectively classified PC patients into low- and high-risk categories across all three cohorts (test1, test 2, and test 3) (**Supplementary Figures 2B–D**). Notably, the median risk score functioned as a benchmark in the train dataset when separating the samples in test1, test2, and test3. In the three cohorts, similarities were observed between the risk score distributions and survival status recorded in the train set and those reported in the internal (test1 and test2 cohorts) and the external validation dataset (**Supplementary Figures 2F–H**). The expression patterns of the MTGs-OS reported by heatmaps in the test1, test2, and test3 cohorts were similar to that in the train cohort (**Supplementary Figures 2G–L**).

### 3.8 The discrepancy in drug sensitivity between low and high-risk patients

**Figure 12:**
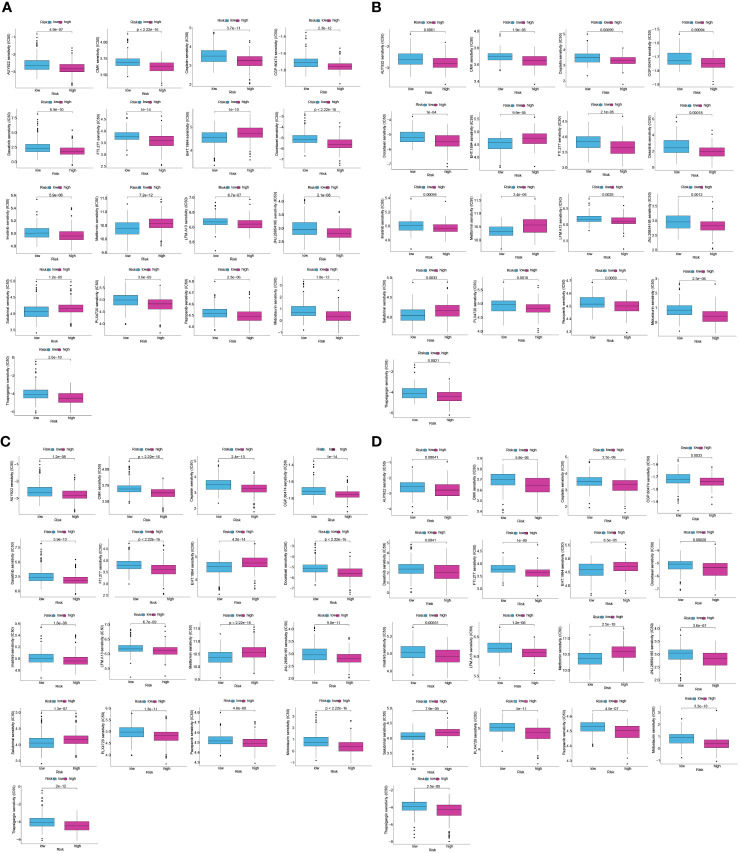
Difference in drug sensitivity between low and high-risk patients in train, test1, test2, and test3 cohorts. The box plot for the estimated IC50 of 12 different types of regularly used chemotherapy regimens conducted in **(A–D)**, **(A–D)** for low-risk (blue) and high-risk patients (purple). The 17 types of chemotherapeutic agents are AUY922, CGP.60474, cisplatin, CMK, dasatinib, docetaxel, EHT.1864, FTI.277, imatinib, JNJ.26854165, LFM.A13, metformin, midostaurin, pazopanib, PLX4720, salubrinal, and thapsigargin. On top of each cluster, the corresponding p-values are shown, and the P< 0.05 denotes the significance level.

As mentioned above, targeted therapies have shown promise in the management of PC, and this is becoming increasingly important. Hence, to identify potentially sensitive medications in both high- and low-risk patients, the “Wilcox.test” tool was used. To be selective, a drug had to show statistical significance across all four categories (train, test1, test2, and test3); we found that 17 distinct drugs had different sensitivities for all the cohorts (**Figures 12A–D**). The 17 drugs were listed as follows: AUY922, CGP.60474, cisplatin, CMK, dasatinib, docetaxel, EHT.1864, FTI.277, imatinib, JNJ.26854165, LFM.A13, metformin, midostaurin, pazopanib PLX4720, salubrinal, and thapsigargin.

### 3.9 The discrepancy in immune cell infiltration and immune checkpoint genes between low- and high-risk patients

**Figure 13:**
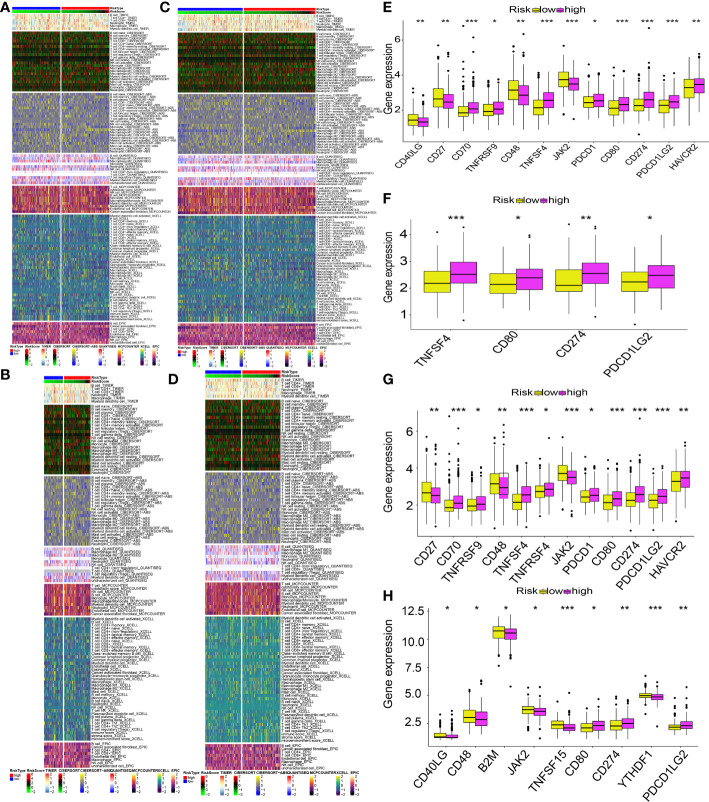
MTG-RPS-based analysis of tumor immune microenvironment. **(A–D)** Comparisons of low- and high-risk groupings in the train, test1, test2, and test3 cohorts revealed distinct patterns of immune cell infiltration, as shown by the heat maps. **(E–H)** Immune checkpoint expression varied across low- and high-risk groupings throughout the train, test1, test2, and test3 cohorts, as depicted by the box plots. (* designates p<0.05; ** signifies p< 0.01; *** designates p< 0.001).

The different levels of immune cell infiltration in the low- and high-risk patients were evaluated according to the TIMER, CIBERSORT, CIBERSORT-ABS, QUANTISEQ, MCPCOUNTER, XCELL, and EPIC algorithms. For both derivation and validation cohorts, the distribution of immunocyte infiltration exhibited obvious discrepancy between high- and low-risk category, further implying the crucial role of mitochondria genes associated with oxidative stress in tumor immune microenvironment (**Figures 13A–D**). In addition, we also evaluated the levels of expression of ICGs in high- and low-risk patients, given the significance of ICGs in the anti-tumor activity of immune cells. All of the ICGs in the train set exhibited substantial differences, as our findings showed (**Figure 13E**). In the test1 datasets, there was a substantial difference in four ICGs between low- and high-risk categories (**Figure 13F**). The findings of the immune checkpoints performed on the test2 cohorts were comparable to those performed on the train cohorts (**Figure 13G**). There were nine ICGs that substantially expressed differing amounts in the low- and high-risk patients in the test3 cohorts (**Figure 13H**). Even though the number of genes with differential expression varied across the training and validation sets, the expression pattern of nearly all ICGs was reduced in the low-risk category in contrast to the high-risk category. These findings might explain why the low risk patients had a favorable prognosis.

### 3.10 Cluster analysis for 226 PNET patients based on MTGs-OS scores

After performing cluster analysis, 226 patients with PNET were successfully stratified into three clusters (i.e. C1, C2, and C3) (**Supplementary Figure 3A**). Both C1 and C2 clusters demonstrated relatively higher MTGs scores than C3 cluster; however, there is no significant difference of MTGs scores between C1 and C2 clusters (**Supplementary Figure 3B**). Thus, C1 and C2 clusters with similar MTGs scores were subsequently integrated and defined as S1 subtype. The C3 cluster with relatively lower MTGs scores was regarded as S2 subtype. Likewise, significantly higher MTG scores were observed in PNET patients with S1 subtype than S2 subtype (**Supplementary Figure 3C**). In addition, we compared the expression profiles of oncogenes and tumor suppressor genes between S1 and S2 subtypes (**Supplementary Figure 4**). Of note, the expression levels of GNAS, HRAS, PDZRN3, and SYNE2 showed significantly different between two subtypes. More importantly, we also compared the discrepancies in metabolism and immune-related pathways between two subtypes. Our findings showed that the S1 subtype was accompanied by higher activities of sphingolipid metabolism and lower activities of B cell and T cell receptor signaling pathway (**Supplementary Figure 5**).

## 4 Discussion

Pancreatic cancer is a type of cancer that begins in the pancreas. It is one of the most aggressive and deadly forms of cancer with a five-year survival rate of just 8% in the United States (33). And it is the sixth leading cause of cancer-related mortality in China (34). As we all know, the pancreas is the largest endocrine gland in the body. When the hormone-producing pancreatic cells, such as islet A cells and B cells and so on, become cancerous, which could lead to the PNET (35). And the incidence of PNET is increasing in the past decades and account for 3%-7% of pancreatic tumors (35). The MDF can lead to mitochondrial OS producing excessive ROS, which could contribute to base replacement mutations of T/C or G/A in mtDNA. Therefore, the mtDNA mutation is related to the oxidative stress environment caused by the MDF, which is carcinogenesis (36, 37). Additionally, the MDF can lead to an increase in pancreatitis, which can also increase the risk of pancreatic cancer (38–40). Researches revealed that MDF has been linked to an increased risk of pancreatic cancer (41–44). Similarly, the functions of hormone-producing pancreatic cells depend on the mitochondria, so the MDF may affect the normal function of pancreatic cells leading to the occurrence of PNET (45, 46). At present, patients with PC has poor prognosis and strong individual heterogeneity. Nevertheless, there is no study investigating the omics of mitochondrial disorders and oxidative stress to identify individual differences in patients with PC and PNET. Consequently, we evaluated the activity of MTGs-OS in each patient based on a series of bioinformatics algorithms, which are beneficial to the development of personalized treatment strategies for patients with PC and PNET.

First, we identified 469 differentially expressed MTGs-OS guided by the MitoCarta3.0 and GeneCard database using the traditional method of difference analysis. Notably, key genes in disease pathogenesis often have both variable expression patterns and high clinical relevance. Therefore, we discovered 56 differentially expressed MTGs-OS between PC and normal pancreatic tissues and correlating with PC prognoses. In addition, MTGs-OS were shown to be protective factors against PC. We also illustrated the co-expressed relationship among the 56 MTGs-OS. We discovered that all genes have at least one interaction with each other, with most of these interactions being positively correlated.

The expression features, prognostic implications, mutation profiles, methylation patterns, and signaling pathway correlations of MTGs-OS were then investigated in various human tumors using bioinformatics-related methods for the first time. For instance, ALDH1L1 regulates the overall flow of one carbon group in the folate-dependent biosynthetic pathway. Through promoter methylation, it is significantly and frequently down-regulated in malignant tumors (47–49). Recent research has highlighted the enzyme’s potential as a marker of invasive carcinoma and as a tumor suppressor, which was in accordance with our bioinformatics analyses (49).

According to the MTGs-OS, we plotted the pan-cancer map for the first time, which provided novel insights and preliminary research foundation into the involvements of MTGs-OS in pan-cancer. Considering the importance and relevance for MTGs-OS in pan-cancer, we could try to classify the human tumors based on the MTGs-OS. And MTGs-OS identification might help translate specific immunotherapies for one type of cancer into patients with other types of cancer. At the same time, the pan-cancer map might provide the guidance for the basic scientific research exploration of the MTGs-OS in the future. For patients with PC, we discovered that most MTGs-OS existed as putative protective genes. This result is consistent with the earlier studies (50–52), which show that appropriate upregulation of MTGs-OS might contribute to improved patients’ prognoses in PC. The mitochondrial ROS were shown to be beneficial to PC, which could provide new insights and points for exploring the mechanism of action of MTGs-OS in fundamental researches and treating the patients with PC in clinical.

We then classified PC patients into three clusters according to their MTGs-OS expression. Patients with PC who belonged to the MTGs-OS inactive cluster had a worse survival rate compared to those belonging to the MTGs-OS active cluster. Similar to these findings, we discovered that MTGs-OS were mostly protective.

At present, targeted drugs exist for several neoplasms, including breast and ovarian malignancies, but not for PC (53). Considering the crucial effects of MTGs-OS in PC, we explored the roles of several commonly used drugs that target MTGs-OS in the treatment of PC. We found that the three clusters responded differentially depending on the drug selected. If the levels of MTGs-OS expression were utilized as the gold standard for classifying PC, each patient with the disease may get a more individualized and effective course of therapy. Patients with inactive MTGs-OS, for instance, could respond better to AICAR(AMPK activator) and cisplatin, gemcitabine, AZD6482(PI3Kβ inhibitors), and so on.

Notably, the balance between tumor suppressor genes and oncogenes may exert a role in the onset and advancement of PC and related patient prognosis. By disrupting the balance between oncogenes and tumor suppressor genes, MTGs-OS may improve the prognoses of PC individuals. For example, the increased levels of oncogenes expression (STAB1, TENM3, PIK3CA, ZFHX4, ADAMTS12, GLI3, HMCN1) and attenuated expression of tumor suppressors, such as RNF4, in the C3 cluster were correlated with dismal prognoses. At the same time, we also found that various metabolism and immune-related pathways exhibited significant differences in the 3 clusters. Most of the protective metabolism pathways such as KEGG_GLYCINE_SERINE_AND_THREONINE_METABOLISM, were inactive in C3 contributing to the worse prognosis for PC individuals. Interestingly, almost all the immune-associated pathways were more active in C3, indicating the degree of malignancy for PC was highest in C3.

Immune cells, which are part of the TME, contribute to tumor clinical outcomes and hence represent potential therapeutic targets (54, 55). Consequently, we examined the link between immune cell infiltration and MTGs-OS. Our findings illustrated that MTGs-OS performs an essential function in immune activation, which suggests that the MTGs-OS-inactive subgroup of PC has a higher grade of malignancy. Further, we discovered that MTGs-OS can trigger both primary and secondary resistance to immune checkpoint activators. As a result of its overexpression of immunological checkpoints, the C3 cluster (MTGs-OS-inactive) likely has the lowest anti-tumor immunity. This may explain the worse clinical outcomes for patients in MTGs-OS-inactive. Treg cells, mast cells, and the T helper cell might enhance the development of neoplasm and promote tumor metastasis resulting in a poor prognosis in PC (56–58). The data show that patients in the C3 subtype have the poorest prognosis, which may be attributed to a negative correlation between immune cells that promote the malignancy and the MTGs-OS score. Our study identified the molecular inhibition of MTGs-OS in patients with PC and revealed the different characteristics in the 3clusters, such as the expression of tumor suppressor genes and oncogenes, the activation of vital pathway and TME and so on. In addition, according to the characteristic, it is beneficial to carry out personalized intervention for PC patients in clinical and promote the research development of PC.

Based on the results above, we could consider the MTGs-OS play an important role in the development of PC. However, because of the MTGs-OS molecular heterogeneity complex function of each MTGs-OS, the classification cannot be able to accurately calculate the clinical outcome of each patient. Therefore, we established a molecular prognostic signature that can accurately predict the clinical outcomes in patients with PC. We used the LASSO-Cox regression analysis to formulate a prognostic signature consisting of 11 MTGs-OS that can be applied in the prediction of PC patients’ overall survival status. The 11 hub MTGs-OS were BIK, COQ4, MCRIP2, SOD1, NDUFB8, MRPL50, MRPL14, RFK, NMNAT3, BNIP3L, MRPS5. The MTGs-RPS we established could predict the prognosis of PC stably and reliably, which was verified by the internal validation and external validation sets. The MTGs-RPS might help the clinical doctors to choose the suitable and personal treatment therapy for patient with PC. Furthermore, we found the variations in the expression of the 11 hub MTGs-OS between PAAD and normal pancreatic samples by bioinformatics. We verified the reliability of the bioinformatics analysis results by qRT-PCR in five kinds of cell lines(vitro) and IHC(vivo) in the HPA database. Almost the validated results were consistent with those predicted by the bioinformatics analysis.

The similar function of the 11 hub genes in tumor or pancreatic cancer has been reported in other researches. BIK is a proapoptotic gene, there were research showing liposome complexed with mutant Bik(T33D/S35D) gene could enhance the anti-tumor effect of BIK in various animal cancer models, including the pancreatic cancer (59). SOD1 could eliminate toxic radicals. Study show the SOD1 could accelerate the invasive and migratory of pancreatic cancer by the H2O2/ERK/NF-κB axis and regulate the expression of EMT-related genes (60). Moreover, accumulating evidence have shown that the expression of SOD1 is significantly up-regulated in many types of malignant tumors, which may lead to the deterioration of the disease and poor prognosis by regulating cell proliferation and oxidative stress (61). As for BNIP3, demethylation of BNIP3 with a methyltransferase inhibitor restores gene expression and induces hypoxic-mediated cell death. We can consider the BNIP3 as a potential target for new therapies aimed at treating pancreatic cancer (62). And MRPS5 could help in protein synthesis within the mitochondrion. The knockdown of MRPS5 obviously could inhibit the pancreatic cell lines proliferation (63). NMNAT3 might play a positive role in the immunotherapy treatment of tumors by suppling NAD+ precursors (64). And NDUFB8 participate in the constitute of mitochondrial respiratory chain complex I. Lots of researches revealed the NDUFB8 is a potential target in many kinds of human tumors, such as cervical cancer tissue, gastric cancer, breast cancer, colorectal cancer, nasopharyngeal carcinoma and glioblastoma (65–70), nevertheless, no study explored the relationship between the NDUFB8 and pancreatic cancer. COQ4 is the component of the coenzyme Q biosynthetic pathway, the COQ4 mutations might lead to early-onset mitochondrial diseases (71). MRPL14 is part of 2 intersubunit bridges in the assembled ribosome, which might be involved in the occurrence and development of thyroid tumors (72). RFK is essential for TNF-induced ROS production, which can influence the progress of breast invasive carcinoma and human prostate cancer (71, 73). However, the role of COQ4, MCRIP2, MRPL50, MRPL14, RFK, and NMNAT3 in pancreatic tumor has not been reported.

Based on the prognostic signature, we grouped the patients with PC into high-risk and low-risk categories by using bioinformatics technology. The findings of KM analysis illustrated that the high-risk patients exhibited a shorter overall survival compared to the low-risk patients. Based on the ROC curve, it was determined that the MTGs-RPS was highly accurate in anticipating 5-year survival rates. Seventeen different types of drugs were evaluated across both the training and validation datasets. All 17 drugs were more effective for the patients with PC in the high-risk subgroup, which might guide the clinical treatment. The scientificity, reliability, and precision of MTGs-RPS were confirmed in the training, internal validation, and external validation sets. Independently, our MTGs-RPS was able to predict the prognosis in PC, hence it may be used as a prognostic marker in PC.

Given the significance of immune infiltration in PC, we investigated its potential functions in the MTGs-RPS in further depth. The study indicated that high- and low-risk patients had different patterns of immune cell infiltration. We discovered that the high-risk patients had an immunosuppressive phenotype, which is only accompanied by a multitude of M0 macrophage infiltration; conversely, the low-risk subgroup was named active anti-tumor immune with many antitumor immune cells (e.g. CD4+ T cells, CD8+T cells, B cells). Additionally, the varied clinical results might be attributed to the varying expression levels of immune checkpoints.

Consider that most PNETs are malignant and more than 60% of patients have tumor metastasis when diagnosed, which is accounting for 3%-7% of PC (74, 75), we also explored the function of MTGs-OS in PNET. Similar as in PC, 226 patients with PNET were separated into 3 clusters, named C1, C2 and C3. And patients in C1 and C2 were included in S1 subtype because of semblable high MTGs-OS scores. Patients in C3 were low MTGs-OS scores named S2. For patients in S1 represented the high MTGs-OS scores, the activation of sphingolipid metabolism and inhibition of B cell and T cell receptor signaling pathway and the high expression of oncogenes(especially for GNAS, HRAS, PDZRN3, and SYNE2) were more active than that in S2 subgroup. The results revealed the activation level of MTGs-OS also play important role in PNET, which might predict prognosis of patients with PNET innovatively and precisely in further research. More important, our findings might provide novel target in the MTGs-OS for tumor therapy improving the outcomes of patients with PC and PNET.

There are a several limitations that should be mentioned in the study. Firstly, the novel prognostic model was both constructed and validated with retrospective data from public. More prospective real-world data are need to warrant to validate the clinical utility of the MTGs-RPS. Secondly, further mechanism investigations are needed to explain the role of the 11 MTGs-OS in the occurrence and progression of PC and PNET. Thirdly, the multi-omics data of PC and PNET did not provide the clinical information, such as the tumor recurrence and metastasis data. Neither the classification nor the MTGs-OS could provide valuable guidance and help for tumor recurrence and metastasis. Despite the limitations, the advantages and clinical significance of our results cannot be ignored. Our research could still provide guidance for MTGs-OS -related basic research of and clinical treatment of PC and PNET.

## 5 Conclusion

We systematically investigated the molecular properties of MTGs-OS and its prognostic potential, given the critical role of mitochondria and the varied effects of OS and their interplay in PC. Furthermore, abnormal MTGs-OS activity was linked to the oncogenesis of PC and PNET in the first place. In addition, the activation of MTGs-OS served as a protective factor for PC. Moreover, patients with PC can be classified into three clusters (i.e. MTGs-OS-normal, MTGs-OS-active, and MTGs-OS-inactive) with different characteristics of prognosis, immune characteristics, and drug sensitivity. And patients with PNET were successfully stratified into 2 subtypes(S1 and S2). The different characteristics for expression of oncogenes and tumor suppressor genes and immune and metabolism pathway between S1 and S2 subtypes were compared. Finally, we construct a novel MTGs-RPS signature that provides a superior prognostic prediction for PC patients. In addition to enhancing the treatment plan, the signature will provide individual direction for clinical medication use, making it highly effective for patients with PC. In conclusion, the research might offer a novel strategy to predict the prognosis provide clinical guidance for the treatment of PC.

## Data availability statement

The original contributions presented in the study are included in the article/**Supplementary Material**. Further inquiries can be directed to the corresponding authors.

## Author contributions

Each author bears full responsibility for the content and writing of the manuscript. Collectively, the authors made significant contributions to the study’s conception, data gathering and analysis, manuscript writing, and manuscript revision. All authors contributed to the article and approved the submitted version.

## Glossary

**Table:**

Gene	full terms
PDK4	Pyruvate Dehydrogenase Kinase
ALDH1L1	Aldehyde Dehydrogenase 1 Family Member L1
ACACB	Acetyl-CoA Carboxylase Beta
EPHX2	Epoxide Hydrolase 2
GATM	Glycine Amidinotransferase, Mitochondrial
ACAT1	Acetyl-CoA Acetyltransferase 1
ETFDH	Electron Transfer Flavoprotein Dehydrogenase
GLDC	Glycine Decarboxylase
ACSF2	Acyl-CoA Synthetase Family Member 2
CYP27A1	Cytochrome P450 Family 27 Subfamily A Member 1
ACSS1	Acyl-CoA Synthetase Short Chain Family Member 1
AKR1B10	Aldo-Keto Reductase Family 1 Member B10
POLG2	DNA Polymerase Gamma 2, Accessory Subunit
NDUFB8	NADH:Ubiquinone Oxidoreductase Subunit B8
MRPS22	Mitochondrial Ribosomal Protein S22
STAB1	Stabilin 1
TENM3	Teneurin Transmembrane Protein 3
PIK3CA	Phosphatidylinositol-4,5-Bisphosphate 3-Kinase Catalytic Subunit Alpha 2
ZFHX4	Zinc Finger Homeobox 4
ADAMTS12	ADAM Metallopeptidase With Thrombospondin Type 1 Motif 12
GLI3	GLI Family Zinc Finger 3
HMCN1	Hemicentin 1
RNF43	Ring Finger Protein 43
PTEN	Phosphatase And Tensin Homolog
NDUFA7	NADH:Ubiquinone Oxidoreductase Subunit A7
NDUFA3	NADH:Ubiquinone Oxidoreductase Subunit A3
MMAB	Metabolism Of Cobalamin Associated B
MCRIP2	MAPK Regulated Corepressor Interacting Protein 2
COQ4	Coenzyme Q4
UCP2	Uncoupling Protein 2
ACAT1	Acetyl-CoA Acetyltransferase 1
DLAT	Dihydrolipoamide S-Acetyltransferase
FTH1	Ferritin Heavy Chain 1
MPV17	Mitochondrial Inner Membrane Protein MPV17
PMAIP1	Phorbol-12-Myristate-13-Acetate-Induced Protein 1
PPTC7	Protein Phosphatase Targeting COQ7
GLDC	Glycine Decarboxylase
SOD1	Superoxide Dismutase 1
MRPL50	Mitochondrial Ribosomal Protein L50
BIK	BCL2 Interacting Killer
MRPL14	Mitochondrial Ribosomal Protein L14
RFK	Riboflavin Kinase
NMNAT3	Nicotinamide Nucleotide Adenylyltransferase 3
BNIP3L	BCL2 Interacting Protein 3 Like
MRPS5	Mitochondrial Ribosomal Protein S5
GNAS	GNAS Complex Locus
HRAS	HRas Proto-Oncogene, GTPase
PDZRN3	PDZ Domain Containing Ring Finger 3
SYNE2	Spectrin Repeat Containing Nuclear Envelope Protein 2
MTGs	mitochondrial genes
OS	oxidative stress
PC	pancreatic cancer
PAAD	pancreatic adenocarcinoma
PNET	pancreatic neuroendocrine tumor
MTGs-OS	mitochondrial genes related to oxidative stress
MTGs-RPS	MTGs-related prognostic signature
mtROS, ROS/RNS	reactive oxygen/nitrogen species
MDF	mitochondrial dysfunction mitochondrial reactive oxygen species
DDR	DNA damage and repair
MTGs-OS	mitochondrial genes related to oxidative stress
TME	Tumor microenvironment
OS	Overall survival
ssGSEA	Single sample gene set enrichment analysis
GSEA	Gene set enrichment analysis
GEO	Gene Expression Omnibus
GTEx	Genotype-Tissue Expression
ICGC	International Cancer Genome Consortium
TCGA	The Cancer Genome Atlas
GEPIA	Gene Expression Profiling Interactive Analysis
FPKM	Fragments Per Kilobase Million
TPM	Trusted Platform Module
GSVA	Gene Set Variation Analysis
IHC	immunohistochemistry
qRT-PCR	Quantitative real-time PCR
(ATCC, Manassas, VA, USA)	The American Type Culture Collection
DMEM	Dulbecco's modified Eagle's medium
FBS	Fetal bovine serum
IMDM	Iscove&rsquo;s Modified Dulbecco medium
HPA	Human Protein Atlas
GDSC	Genomics of Drug Sensitivity in Cancer
SIRTs	Sirtuins
HDACs	Histone deacetylases
ICGs	immune checkpoints genes
Treg	Regulatory T cells
LncRNAs	Long non-coding RNAs
Gtf	Gene transfer format
LASSO	Least absolute shrinkage and selection operator
AUC	Area under the curve
ROC	Receiver-operating characteristic
TMB	Tumor mutation burden
CNV	Copy number variations
SNV	Single-nucleotide variant
UCEC	Uterine corpus endometrial carcinoma
SKCM	Skin cutaneous melanoma
STAD	Stomach adenocarcinoma
COAD	Colon adenocarcinoma
LUSC	Lung squamous cell carcinoma
LUAD	Lung adenocarcinoma
