# Supplementary figures and images for: Determination and characterization of molecular heterogeneity and precision medicine strategies of patients with pancreatic cancer and pancreatic neuroendocrine tumor based on oxidative stress and mitochondrial dysfunction-related genes
Source: Front Endocrinol (Lausanne). 2023 May 8;14:1127441. doi: 10.3389/fendo.2023.1127441 (PMC10200886; doi:10.3389/fendo.2023.1127441)

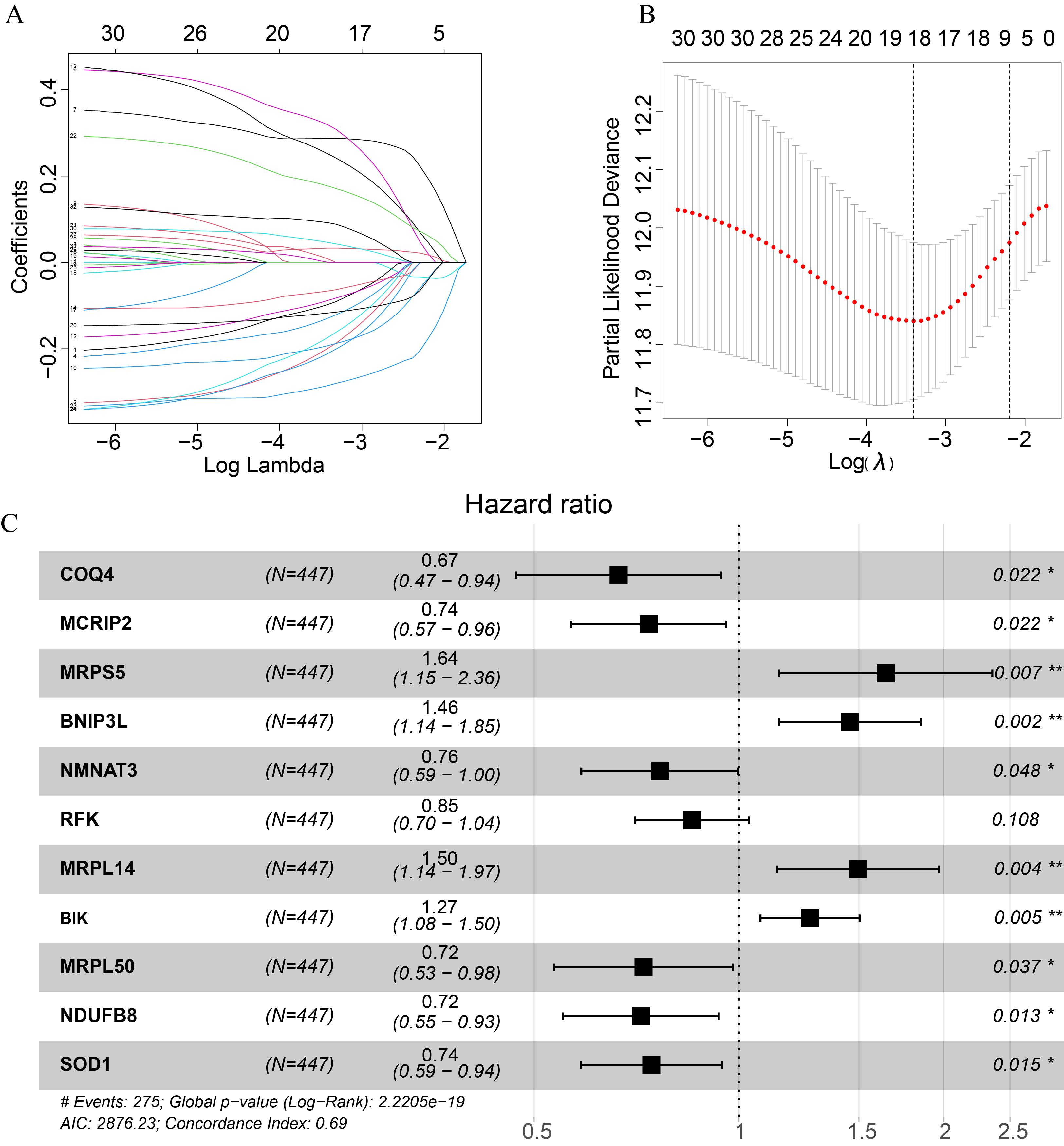

Supplement: Supplementary file 1 [file Image_1.jpeg]

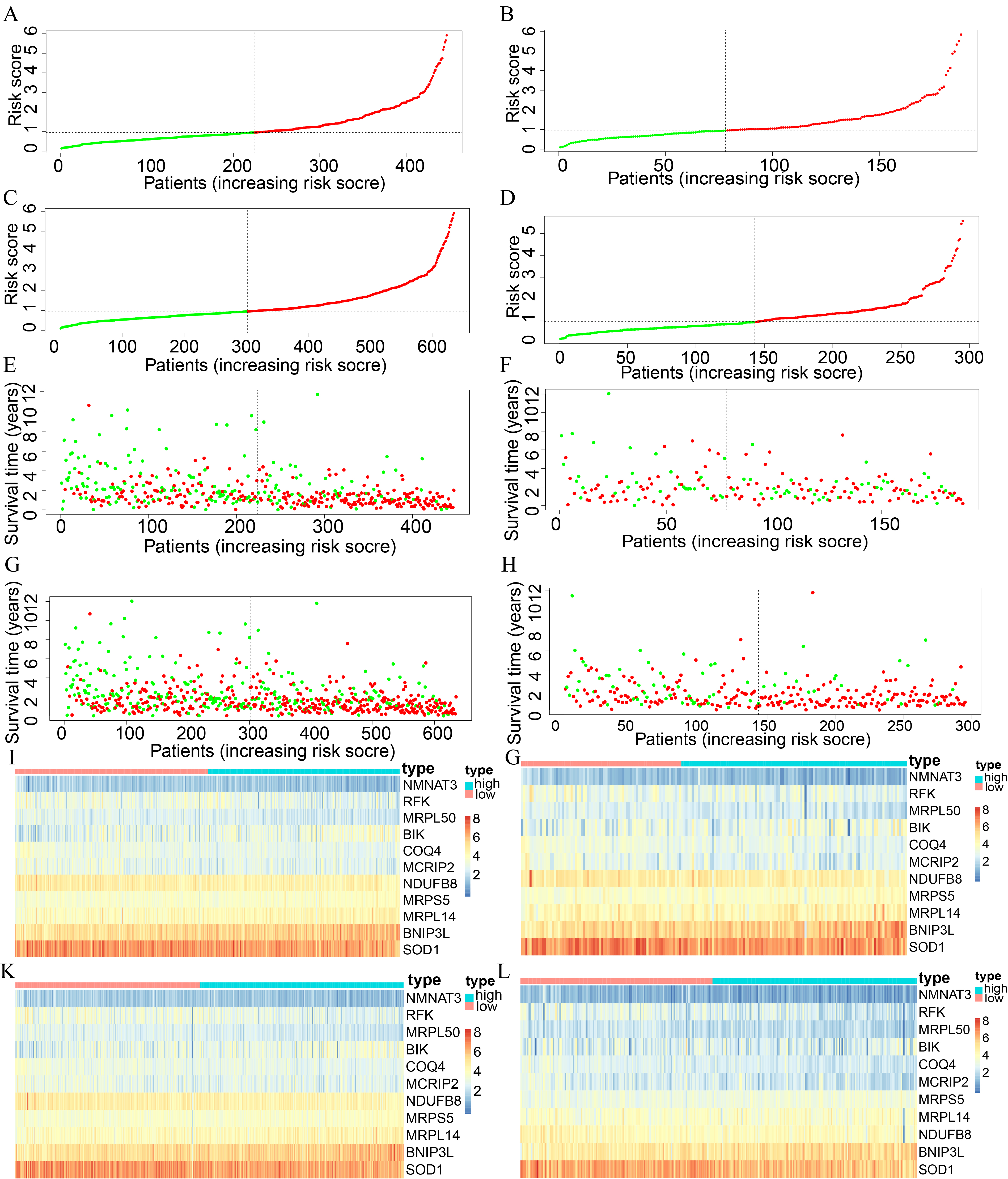

Supplement: Supplementary file 2 [file Image_2.jpeg]

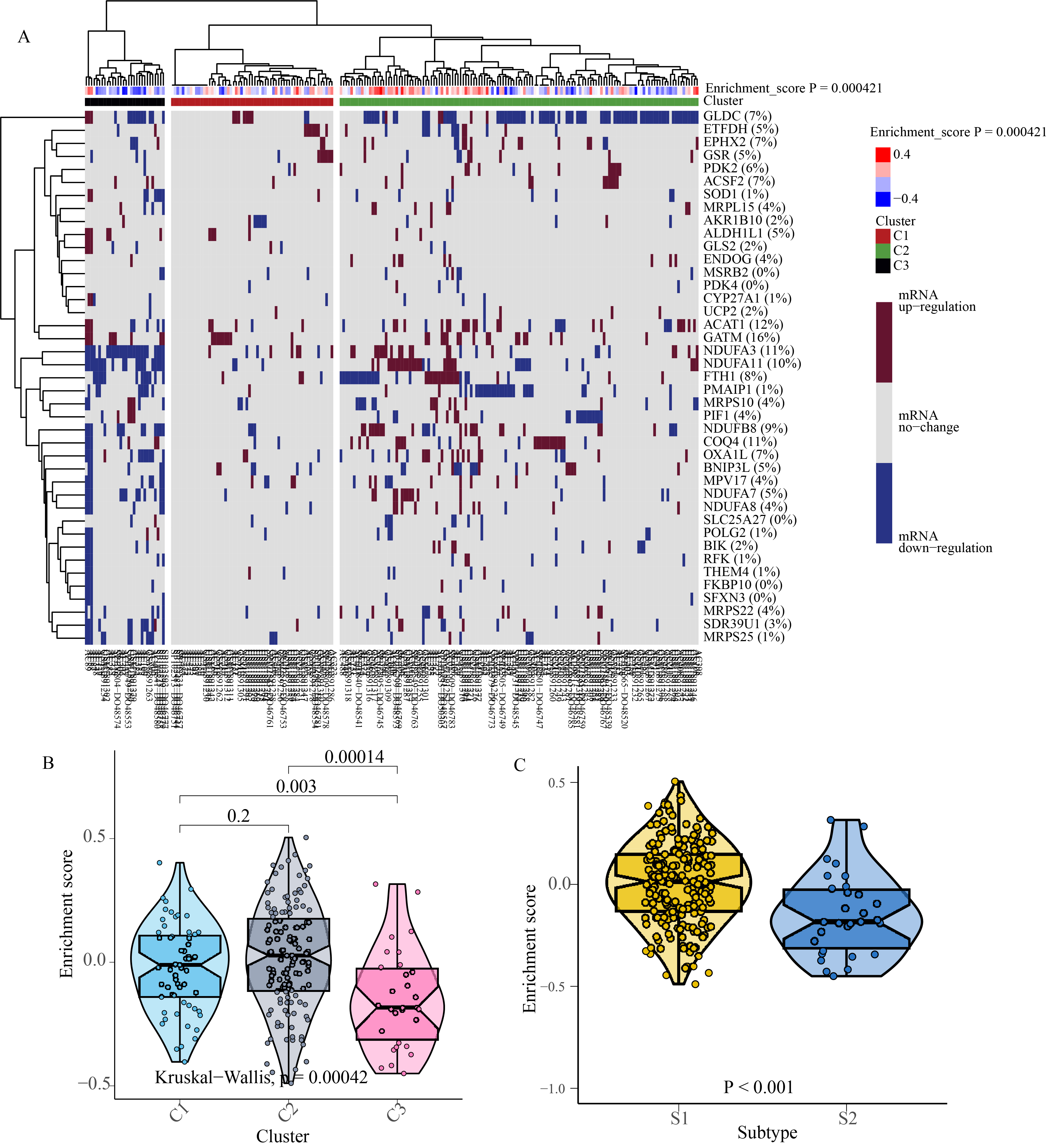

Supplement: Supplementary file 3 [file Image_3.jpeg]

S1

S2

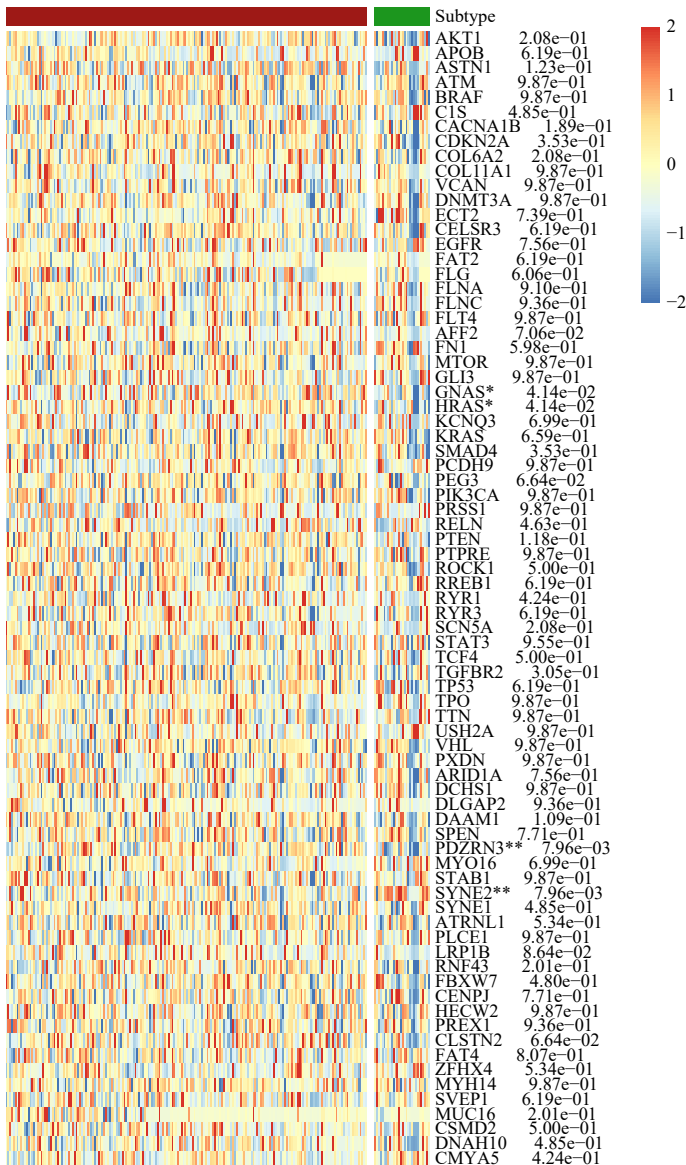

Supplement: Supplementary file 4 [file Image_4.pdf]

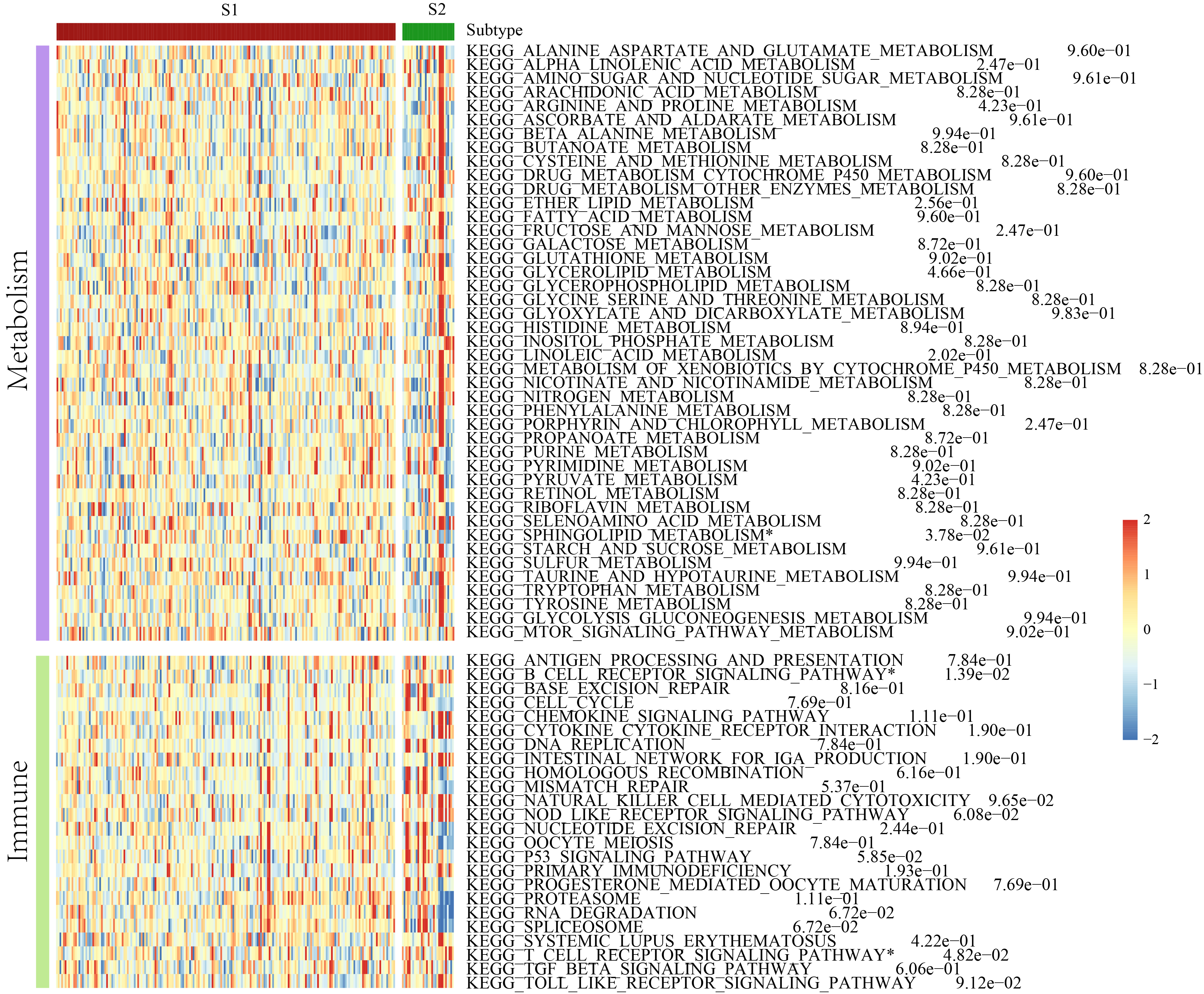

Supplement: Supplementary file 5 [file Image_5.jpeg]
